# Immunosuppression in Older Kidney Transplant Recipients: A Randomized Controlled Trial

**DOI:** 10.1681/ASN.0000000924

**Published:** 2025-11-07

**Authors:** Jan-Stephan F. Sanders, Silke E. de Boer, Jip Jonker, Frederike J. Bemelman, Michiel G.H. Betjes, Aiko P.J. de Vries, Luuk Hilbrands, Marc Hilhorst, Dirk R.J. Kuypers, Priya Vart, Arjan D. van Zuilen, Dennis A. Hesselink, Stefan P. Berger

**Affiliations:** 1 Division of Nephrology, Department of Internal Medicine, University Medical Center Groningen, University of Groningen, Groningen, The Netherlands; 2 Renal Transplant Unit, Amsterdam UMC, University of Amsterdam, Amsterdam, The Netherlands; 3 Division of Nephrology and Transplantation, Department of Internal Medicine, Erasmus MC Transplant Institute, Erasmus MC, Rotterdam, The Netherlands; 4 Division of Nephrology, Department of Internal Medicine, Leiden Transplant Center, Leiden University Medical Center, Leiden University, Leiden, The Netherlands; 5 Department of Nephrology, Radboud University Medical Center, Nijmegen, The Netherlands; 6 Department of Nephrology and Renal Transplantation, University Hospitals Leuven, Leuven, Belgium; 7 Department of Nephrology, University Medical Center Utrecht, Utrecht University, Utrecht, The Netherlands

**Keywords:** clinical trial, immunosuppression, kidney transplantation, survival analysis, tacrolimus

## Abstract

**Key Points:**

- Low-dose tacrolimus, everolimus, and prednisolone did not result in a higher rate of successful transplantation in older kidney transplant recipients.
- Low-dose tacrolimus, everolimus, and prednisolone did not result in better kidney function or fewer infections in older kidney transplant recipients.

**Background:**

We hypothesized that older kidney transplant recipients receiving low-dose tacrolimus, everolimus, and prednisolone (TEP) have better outcomes than patients receiving standard-dose tacrolimus, mycophenolate mofetil, and prednisolone (TMP).

**Methods:**

The OPTIMIZE study was a randomized clinical trial in kidney transplant recipients age ≥65 years. Patients receiving a kidney from a deceased donor older than 65 years (stratum A) or a kidney from a deceased donor younger than 65 years or a living donor (stratum B) were included. Patients were randomized to TEP or TMP groups. Tacrolimus target trough levels in the TEP group were 5–7 ng/ml until 3 months, 2–4 ng/ml from 3 to 6 months, and 1.5–4 ng/ml from 6 months onwards. Tacrolimus target trough levels in the TMP group were 8–12, 6–10, and 5–8 ng/ml. Everolimus target trough levels were 3–6 *µ*g/L. The primary end point of successful transplantation was defined as being alive with a functioning graft with an eGFR above a predefined threshold at 2 years after transplantation. Predefined eGFR thresholds were 30 (stratum A) or 45 ml/min per 1.73 m^2^ (stratum B).

**Results:**

A total of 379 patients were randomized, of whom 198 were in stratum A (TEP 97, TMP 101) and 181 in stratum B (TEP 90, TMP 91). The median trough levels for everolimus and tacrolimus were within the target range throughout the study. There was no statistically significant difference in successful transplantation at 2 years between the groups (TEP 94 [50%], TMP 110 [57%]; difference 7% [95% confidence interval, −17 to 3] *P* = 0.91). Regarding the predefined secondary outcomes, patient survival (TEP 167 [89%], TMP 171 [89%]; *P* = 0.95) and graft survival (TEP 155 [83%], TMP 162 [84%]; *P* = 0.65) did not differ significantly. Within strata A and B, there were no significant differences in the end points.

**Conclusions:**

Immunosuppression with low-dose tacrolimus, everolimus, and prednisolone did not result in a higher rate of successful transplantation in *de novo* older kidney transplant recipients compared with immunosuppression with standard-dose TMP.

**Clinical Trial registry name and registration number:**

ClinicalTrials.gov, NCT03797196.

## Visual Abstract

**Figure:**
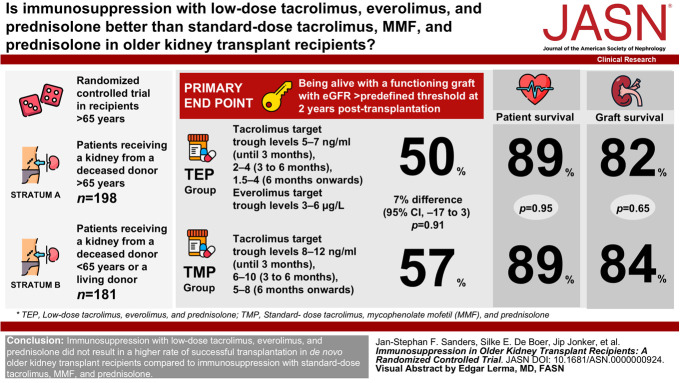

## Introduction

Older patients constitute an increasing proportion of both dialysis and kidney transplant populations. Kidney transplantation in this cohort exhibits distinct, age-related characteristics. Compared with younger recipients, older patients are more frail, have higher comorbidity, and demonstrate markedly different risk profiles.

In younger recipients, graft loss is most commonly attributable to loss of kidney allograft function necessitating return to dialysis or retransplantation. By contrast, among older recipients, death-censored graft loss is relatively uncommon.^1,2^ Instead, outcomes are predominantly determined by patient mortality, with cardiovascular complications, malignancies, and infection-related deaths occurring at higher frequencies.^3–5^ Concurrently, immunosenescence appears to reduce susceptibility to allograft rejection.^1,6^

Furthermore, kidneys procured from older donors are preferentially allocated to older recipients, where diminished allograft function may be the result of preexisting injury and heightened vulnerability to toxicity of calcineurin inhibitors (CNIs).^7^ For older recipients receiving marginal allografts, preservation of graft function by avoiding CNI toxicity and avoidance of over-immunosuppression resulting in less infection might be more important than rejection prevention. The TRANSFORM trial demonstrated that the use of everolimus enabled CNI reduction in *de novo* kidney transplant recipients, with similar rates of rejection and kidney function, and fewer infections up to 2 years post-transplant, compared with standard exposure to CNI.^8,9^ However, in the TRANSFORM study, patients had a mean age of 49 years. Therefore, we hypothesized that the more vulnerable older kidney transplant population, often receiving marginal allografts, would specifically benefit from a regimen of low-dose tacrolimus, everolimus, and prednisolone (TEP) compared with standard-dose tacrolimus, mycophenolate mofetil (MMF), and prednisolone (TMP).

## Methods

The OPen label multicenter randomized trial comparing standard IMmunosuppression with tacrolimus and mycophenolate mofetil with a low exposure tacrolimus regimen In combination with everolimus in *de novo* renal transplantation in Elderly patients (OPTIMIZE) study was an investigator-driven, randomized, multicenter, open-label, intervention trial that was conducted between July 2019 and April 2025. Six Dutch transplant centers and one Belgian transplant center participated in this study. The study protocol was approved by the medical research ethics committee at the University Medical Center Groningen (METC 2018.698) and was registered at www.ClinicalTrials.gov (NCT03797196). The study was conducted according to the Declaration of Helsinki and the International Conference on Harmonization Guidelines for Good Clinical Practice. All patients provided written informed consent. The study protocol and methods were previously published.^10^

### Patients, Randomization, and Study Treatment

The study population consisted of *de novo* kidney transplant recipients age 65 years or older at the time of transplantation. The study consisted of two strata: stratum A included older recipients (≥65 years) of kidneys from older deceased donors (≥65 years) within the Eurotransplant Senior Program. Stratum B included older recipients (≥65 years) of kidneys from living donors (all ages) or deceased donors <65 years. Patients were excluded if there was a high (at discretion of transplant center) or very low immunologic risk (HLA-identical living donor), signs of active infection, or a high chance of unacceptable side effects of the study medication. Detailed inclusion and exclusion criteria can be found in Supplemental Table 1. Participants were enrolled and randomized by treating physicians in the participating centers. Patients were randomized at transplantation in a 1:1 manner using the web-based randomization system ALEA (FormsVision BV, Abcoude, The Netherlands). Randomization was stratified by center in stratum A and separately by donor type (living or deceased) within each center in stratum B.

Two immunosuppressive regimens were tested: a quadruple immunosuppressive regimen with induction therapy with basiliximab, low-dose tacrolimus in combination with everolimus and prednisolone (TEP-group) and the standard therapy consisting of induction therapy with basiliximab, standard-dose TMP-group (Supplemental Figure 1). Tacrolimus target trough levels in the TEP group were 5–7 ng/ml until 3 months, 2–4 ng/ml from 3 to 6 months, and 1.5–4 ng/ml from 6 months onward. Corresponding tacrolimus target trough levels in the TMP group were 8–12, 6–10, and 5–8 ng/ml, respectively. Everolimus target trough levels were 3–6 *µ*g/L throughout the study. Tacrolimus was used as once daily formulation, or as twice daily formulation, when deemed necessary by the local investigator. If tacrolimus was not tolerated, it was allowed to be replaced by ciclosporin. Concomitant medication can be found in the methods paper.^10^

### Outcomes

The primary end point was a binary variable, successful transplantation, defined as survival with a functioning allograft after 2 years with a minimum eGFR of 30 ml/min per 1.73 m^2^ in stratum A and 45 ml/min per 1.73 m^2^ in stratum B. The different kidney function thresholds in the two strata were chosen to account for the lower functional potential of kidneys from older donors in the Eurotransplant Senior Program and based on median eGFR in the recipients within the respective strata an earlier observational study on transplant outcomes in older recipients.^1^ The main secondary end points were the primary end point analyzed separately per stratum and the incidence of treated biopsy-proven acute rejection (tBPAR), classified according to the updated Banff 2019 classification.^11^ The eGFR was assessed according to the CKD Epidemiology Collaboration 2021 creatinine-based calculation at baseline and at day 7, week 4, and months 3, 6, 9, 12, 18, and 24 after transplantation.

### Sample Size

We expected to reach the primary end point (a successful transplantation at 2 years after transplantation) in 48% of the patients in the TMP group. To demonstrate a 14% higher incidence of the primary end point in the TEM group with a power of at least 0.86 and *α* of 0.05, a total sample size of 374 participants (one-sided z-test) was required.

### Statistical Analyses

The primary outcome, successful transplantation, was compared using a z-test for proportions according to an intention-to-treat analysis. In addition, results were analyzed using a generalized mixed-effects model, including treatment as a fixed effect and a random effect for study center and stratum. The interaction between treatment and stratum was tested to assess potential heterogeneity in the effect of the intervention across strata. Patient survival, death-censored graft survival, overall graft survival, and tBPAR were analyzed using a log-rank test. Given the exploratory nature of these analyses, no adjustment was made for multiple testing. Comparison of eGFR at 2 years post-transplantation was done using a *t* test. The eGFR slope per treatment group was estimated using a linear mixed-effects model with time as a fixed effect and random intercepts for stratum and for participants nested within stratum. A similar analysis was performed, with the inclusion of the interaction between time and treatment, to compare eGFR slopes between treatment groups. To assess robustness of these results, in an additional sensitivity analysis, we imputed a value of 0 ml/min per 1.73 m^2^ for eGFR after death-censored graft failure. The incidence of (serious) adverse events was reported as number and percentages and was compared between treatment groups using a chi-square test. Laboratory values of interest (proteinuria, LDL cholesterol, and triglycerides) were compared at 12 and 24 months post-transplantation using a Mann–Whitney *U* test. In the analyses of rates of successful transplantation, a one-sided *P* value is provided, and in all other analyses, a two-sided *P* value is provided. A *P* value < 0.05 was considered statistically significant. All analyses were performed using R version 4.4.1. No interim analysis was performed during the study. A data safety monitoring board performed predefined safety analyses.

## Results

### Patient Population

**Figure 1:**
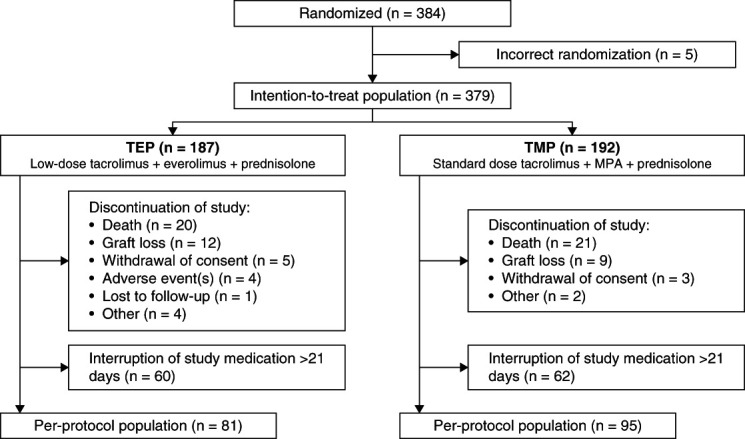
**Study flow chart showing participant disposition.** TEP, tacrolimus, everolimus, and prednisolone; TMP, tacrolimus, mycophenolate mofetil, and prednisolone.

A flow chart of the study enrollment is presented in Figure 1. In total, 379 participants were randomized, of whom 198 were in stratum A and 181 in stratum B. Baseline characteristics of participants are presented in Table 1 for the overall study groups. Recipient and donor characteristics were comparable between the treatment groups. Overall, only 14 (7%) and five (3%) participants discontinued the study prematurely in the TEP and TMP groups, respectively.

**Table 1:** Baseline characteristics of older kidney transplant recipients

Characteristic	TEP (*n*=187)	TMP (*n*=192)
Stratum A, No. (%)	97 (52)	101 (53)
Stratum B, No. (%)	90 (48)	91 (47)
Age, yr	70 (68–73)	70 (67–73)
Female, No. (%)	55 (29)	56 (29)
**Ethnicity, No. (%)**		
Asian	7 (4)	12 (6)
Black	6 (3)	6 (3)
Other	4 (2)	6 (3)
White	170 (91)	168 (88)
BMI, kg/m^2^	26.8±3.5	26.8±4.0
**Kidney disease, No. (%)**		
Diabetes mellitus	34 (21)	28 (16)
Hypertension	25 (16)	31 (18)
Polycystic kidney disease	17 (11)	20 (12)
GN	13 (8)	14 (8)
Glomerulosclerosis	5 (3)	12 (7)
Other	66 (41)	67 (39)
History of treated hypertension, No. (%)	160 (86)	173 (90)
History of malignancy, No. (%)	36 (19)	45 (23)
History of diabetes mellitus, No. (%)	65 (35)	63 (33)
**Donor type, No. (%)**		
Deceased brain death	47 (25)	50 (26)
Deceased circulatory death	85 (46)	90 (47)
Living related	15 (8)	8 (4)
Living unrelated	40 (21)	44 (23)
Donor age, yr	68 (61–71)	68 (58–71)
Prior kidney transplantation, No. (%)	6 (3)	6 (3)
Preemptive transplantation, No. (%)	50 (27)	44 (23)
HLA-A mismatches, 0/1/2 (%)	21/47/33	19/53/29
HLA-B mismatches, 0/1/2 (%)	13/36/51	13/39/48
HLA-DR mismatches, 0/1/2 (%)	13/44/42	12/49/39
Cold ischemia time, h	10.1±5.2	10.5±5.1
**CMV serology, No. (%)**		
D−R−	55 (30)	46 (24)
D−R+	35 (19)	41 (22)
D+R−	35 (19)	44 (23)
D+R+	60 (32)	60 (31)

BMI, body mass index; CMV, cytomegalovirus; D, donor; R, recipient; TEP, tacrolimus, everolimus and prednisolone; TMP, tacrolimus, mycophenolate mofetil and prednisolone.

### Immunosuppression

**Figure 2:**
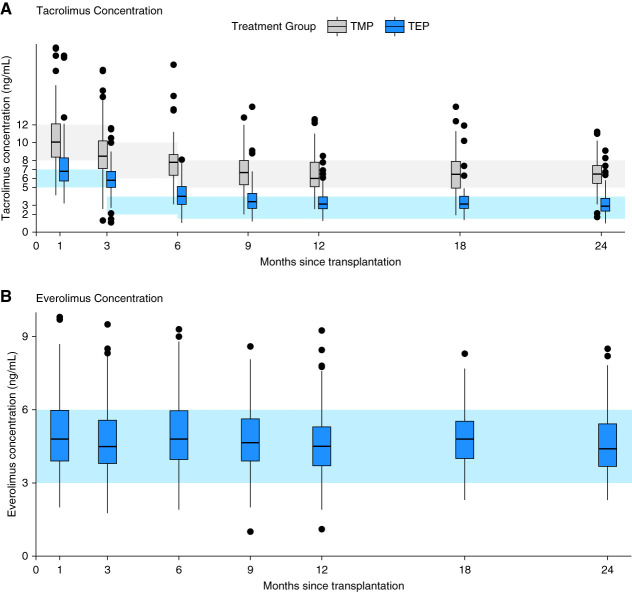
**Tacrolimus and everolimus trough levels.** Exposure to tacrolimus (A) and everolimus (B) according to trough levels at study visits in the TMP and TEP groups. Box and whisker plots depict median values, 25%–75% (boxes) and 1.5 interquartile range from quartile 1 and 3 (whiskers). Predefined ranges for tacrolimus are shown in gray (TMP) and blue (TEP), and for everolimus in blue.

The mean trough levels for tacrolimus and everolimus were mostly within the target range throughout the study (Figure 2 and Supplemental Table 2). The mean tacrolimus trough levels at 3, 12, and 24 months were 5.9±1.7, 3.5±1.3, and 3.3±1.5 ng/ml in the TEP group and 8.7±2.9, 6.5±2.1, and 6.4±1.8 ng/ml in the TMP group, respectively. Discontinuation of study medication was similar between the TEP and TMP groups (43% versus 40%, *P* = 0.59; Figure 1). Most participants continuously received tacrolimus-based immunosuppression, and only a minority of participants switched to ciclosporin (TEP 5, TMP 10). In the TMP group, MMF was maintained at 1000 mg per day in most participants (MMF dose is depicted in Supplemental Figure 2). Interruption of study medication for more than 21 days was mostly related to the occurrence of adverse events (Supplemental Table 3).

### Primary End Point: Successful Transplantation

**Figure 3:**
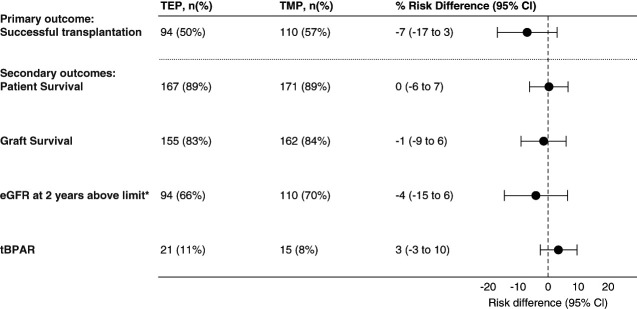
**Absolute risk difference and 95% CI of the primary and secondary study end points between the TEP and TMP treatment groups.** *Proportion calculated in population that had creatinine measurement performed at 2 years post-transplant. CI, confidence interval; tBPAR, treated biopsy-proven acute rejection.

Successful transplantation, defined as being alive with a functioning allograft and an eGFR above the stratum-dependent thresholds at 2 years after transplantation was observed in a total of 204 participants (54%). Of the 187 kidney transplant recipients randomized to the TEP group, 94 (50%) were classified as a successful transplantation as compared with 110 of the 192 kidney transplant recipients (57%) randomized to the TMP group. So, the difference in successful transplantation between the TEP and TMP group was −7% (95% confidence interval [CI], −17 to 3), and the proportion of successful transplantation at 2 years was not significantly higher in the TEP compared with the TMP group (*P*_chi-square_ = 0.91, *P*_mixed-model_ = 0.91; Figure 3). There was also no significant interaction between treatment group and stratum on the outcome of successful transplantation (*P* = 0.82).

### Secondary End Points

**Figure 4:**
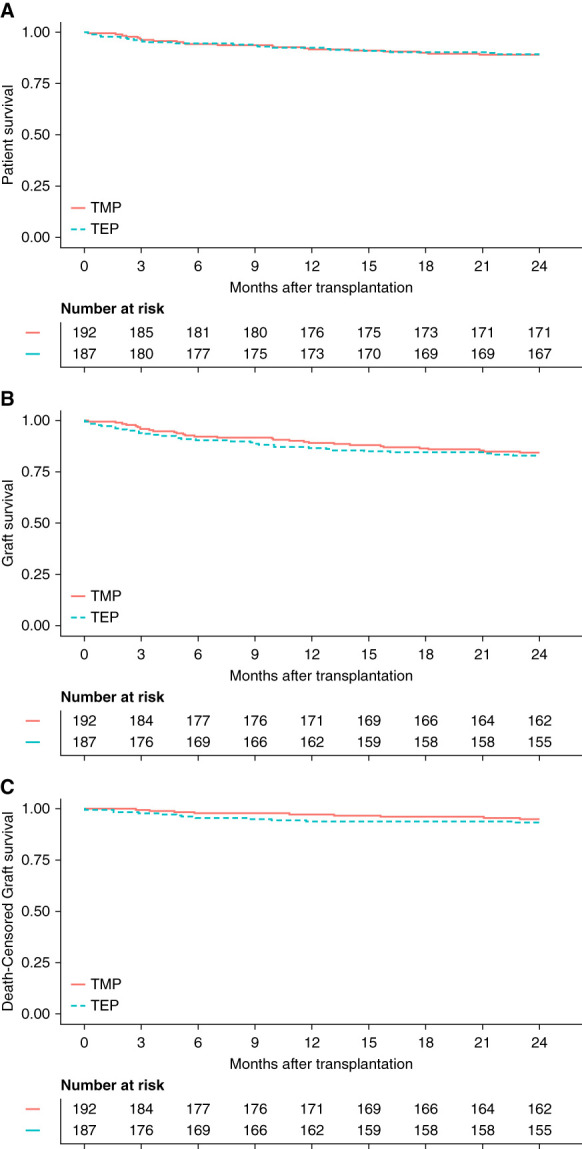
**Patient, graft, and death-censored graft survival.** Kaplan–Meier plot for prespecified secondary outcomes patient survival (A), graft survival (B), and death-censored graft survival (C) in the TEP and TMP treatment groups.

A total of 41 participants died in the first 2 years after transplantation (Figure 4A), without a significant difference in patient survival between the TEP group and the TMP group (TEP 167 [89%], TMP 171 [89%]; difference 0% [95% CI, −6 to 7], *P* = 0.95; Figures 3 and 4A). In stratum A, 14 (14%) participants in the TEP group and 14 (13.9%) participants in the TMP group died (*P* = 0.88). In stratum B, six (7%) participants in the TEP group and seven (8%) participants in the TMP group died (*P* = 0.78).

Graft survival at 2 years after transplantation did not differ significantly between the groups (TEP 155 [83%], TMP 162 [84%]; difference −1% [95% CI, −9 to 6], *P* = 0.65; Figures 3 and 4B). Death-censored graft failure occurred in 12 participants (6%) in the TEP group and in nine participants (5%) in the TMP group (Figure 4C). Death-censored graft survival did not differ significantly at 2 years after transplantation (TEP 93%, TMP 95%, *P* = 0.44). Also, in the two separate strata, there were no significant differences in total graft survival or death-censored graft survival between the TEP and TMP groups.

There was no significant difference in the frequency of tBPAR between the TEP and TMP groups (TEP 21 [11%], TMP 15 [8%]; difference 3% [95% CI, −3 to 10], *P* = 0.22; Figure 3).

### Kidney Function

Kidney function as measured by eGFR was stable from month 1 to month 24 in the intention-to-treat analysis. At month 24, eGFR was 48±19 versus 48±19 ml/min per 1.73 m^2^ in the TEP and TMP group, respectively (*P* = 0.95). Mean eGFR at 24 months also did not differ between treatment groups within stratum A (TEP 42±16 ml/min per 1.73 m^2^; TMP 41±16 ml/min per 1.73 m^2^; *P* = 0.71) and stratum B (TEP 53±20 ml/min per 1.73 m^2^; TMP 55±18 ml/min per 1.73 m^2^; *P* = 0.63).

**Figure 5:**
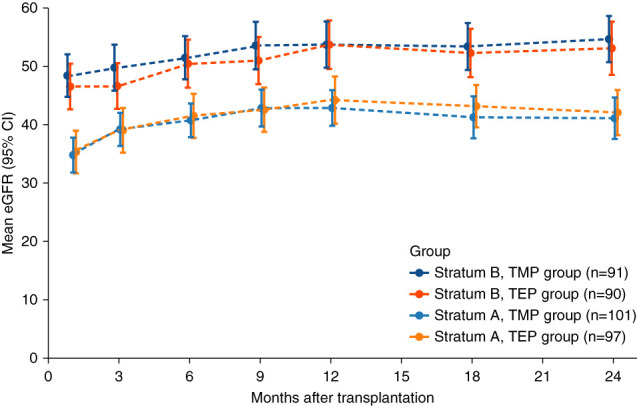
eGFR (mean and 95% CI) over time in the study population stratified by treatment group and stratum, a secondary outcome.

Using a linear mixed-effect model taking into account all available eGFR measurements, the eGFR was estimated to increase 3 ml/min per 1.73 m^2^ (95% CI, 2 to 3) per year in the TEP and 2 ml/min per 1.73 m^2^ (95% CI, 1 to 3) in the TMP group (Figure 5). The difference in eGFR slope was not statistically significant (*P* = 0.35). Also, after imputation of a value of 0 ml/min per 1.73 m^2^ after death-censored graft failure, eGFR slopes did not differ significantly between the TEP and TMP groups (*P* = 0.77).

### Safety Outcomes

Of the 41 deaths, there were 36 cases with a known cause of death. In the TEP group, six participants died of infectious causes, two of malignancies, three of cardiovascular disease, and three had an unknown cause of death. In the TMP group, nine participants died of infection, four died due to a malignant disease, two of cardiovascular disease, and two had an unknown cause of death.

The overall incidences of adverse events (AEs) and serious adverse events were similar between the two arms (Table 2 and Supplemental Table 4). Infections were the most frequent AEs. Incidence of patients with at least one infection did not differ significantly between the TEP (150 [81%]) and TMP groups (158 [83%]; *P* = 0.68). Also, the incidence of viral infections did not differ significantly between the TEP (101, 55%) and TMP group (104, 54%; *P* = 0.98). Specifically, the incidence of coronavirus disease 2019 (COVID-19), cytomegalovirus, and BK virus infection did not differ significantly between the TEP and TMP groups (Table 3).

**Table 2:** Adverse events in older kidney transplant recipients

Adverse Event Category	TEP	TMP	Relative Risk (95% CI)
General disorders and administration site conditions	112 (61%)	116 (61%)	1.00 (0.85 to 1.17)
Renal and urinary disorders	90 (49%)	99 (52%)	0.94 (0.77 to 1.15)
Infections and infestations	149 (81%)	160 (84%)	0.96 (0.87 to 1.06)
Metabolism and nutrition disorders	146 (79%)	145 (76%)	1.04 (0.93 to 1.16)
Vascular disorders	139 (75%)	143 (75%)	1.00 (0.89 to 1.13)
Gastrointestinal disorders	112 (61%)	114 (60%)	1.01 (0.86 to 1.20)
Blood and lymphatic system disorders	99 (54%)	93 (49%)	1.10 (0.90 to 1.34)
Investigations	93 (50%)	92 (48%)	1.04 (0.85 to 1.28)
Nervous system disorders	70 (38%)	89 (47%)	0.81 (0.64 to 1.03)
Respiratory, thoracic, and mediastinal disorders	60 (32%)	65 (34%)	0.95 (0.72 to 1.27)
Immune system disorders	25 (14%)	24 (13%)	1.08 (0.64 to 1.81)
Injury, poisoning, and procedural complications	91 (49%)	99 (52%)	0.95 (0.78 to 1.16)
Surgical and medical procedures	47 (25%)	47 (25%)	1.03 (0.73 to 1.47)
Skin and subcutaneous tissue disorders	37 (20%)	41 (22%)	0.93 (0.63 to 1.38)
Musculoskeletal and connective tissue disorders	58 (31%)	58 (30%)	1.03 (0.76 to 1.40)
Cardiac disorders	53 (29%)	59 (31%)	0.93 (0.68 to 1.27)
Psychiatric disorders	18 (10%)	26 (14%)	0.71 (0.41 to 1.26)
Reproductive system and breast disorders	10 (5%)	11 (6%)	0.94 (0.41 to 2.16)
Neoplasms benign, malignant, and unspecified (incl cysts and polyps)	20 (11%)	35 (18%)	0.59 (0.35 to 0.98)
Eye disorders	14 (8%)	19 (10%)	0.76 (0.39 to 1.47)
Product issues	3 (2%)	5 (3%)	0.62 (0.15 to 2.56)
Endocrine disorders	10 (5%)	3 (2%)	3.44 (0.96 to 12.31)
Surgical and medical procedures	1 (0.5%)	3 (2%)	0.34 (0.04 to 3.28)
Ear and labyrinth disorders	7 (4%)	5 (3%)	1.45 (0.47 to 4.47)
Social circumstances	3 (2%)	0 (0%)	Not applicable
Hepatobiliary disorders	8 (4%)	4 (2%)	2.06 (0.63 to 6.74)
Sexual dysfunction (SMQ)	0 (0%)	2 (1%)	Not applicable
Peripheral sensorimotor neuropathy	1 (0.5%)	0 (0%)	Not applicable
Congenital, familial, and genetic disorders	2 (1%)	2 (1%)	1.03 (0.15 to 7.25)
Injury, poisoning, and procedural complications	0 (0%)	1 (0.5%)	Not applicable
Morganella infection	1 (0.5%)	1 (0.5%)	1.03 (0.07 to 16.38)
Mouth injury	0 (0%)	1 (0.5%)	Not applicable

CI, confidence interval; SMQ, standardized MedDRA query; TEP, tacrolimus, everolimus, and prednisolone; TMP, tacrolimus, mycophenolate mofetil, and prednisolone.

**Table 3:** Adverse events of special interest

Adverse Event Category	TEP	TMP	Relative Risk (95% CI)
**Infection total**	150 (81%)	158 (83%)	0.98 (0.89 to 1.08)
Infection bacterial	107 (58%)	110 (58%)	1.00 (0.84 to 1.19)
Infection viral	101 (55%)	104 (54%)	1.00 (0.83 to 1.21)
*CMV*	18 (10%)	22 (12%)	0.84 (0.47 to 1.52)
*BK virus*	31 (17%)	38 (20%)	0.84 (0.55 to 1.29)
*COVID-19*	53 (29%)	45 (24%)	1.22 (0.86 to 1.71)
Edema or other signs of hypervolemia	110 (59%)	83 (43%)	1.37 (1.12 to 1.67)
**Malignancy total**	16 (9%)	29 (15%)	0.57 (0.32 to 1.01)
Skin malignancy	10 (5%)	13 (7%)	0.79 (0.36 to 1.77)
Malignancy other	7 (4%)	16 (8%)	0.45 (0.19 to 1.07)
Thromboembolic	30 (16%)	26 (14%)	1.19 (0.73 to 1.93)

CI, confidence interval; CMV, cytomegalovirus; COVID-19, coronavirus disease 2019; TEP, tacrolimus, everolimus, and prednisolone; TMP, tacrolimus, mycophenolate mofetil, and prednisolone.

The occurrence of malignancies tended to be lower in the TEP group than the TMP group (TEP 16 [9%]; TMP 29 [15%]; *P* = 0.051), whereas the proportion of participants with skin malignancies (TEP 10 [5%]; TMP 13 [7%]; *P* = 0.57) did not differ significantly between the two treatment groups.

There was a significant difference in edema or other signs of hypervolemia between the TEP (110 [59%]) and TMP groups (83 [43%]; *P* = 0.002). AEs regarding the nervous system tended to occur significantly less frequently in the TEP group (70 [38%]) than in the TMP group (89 [47%]; *P* = 0.09). There were no significant differences in thromboembolic complications between the TEP and TMP groups (*P* = 0.48).

### Laboratory Analyses

At 12 months post-transplantation, urinary protein-creatinine ratio, and serum concentrations of LDL cholesterol and triglycerides were significantly higher in the TEP group (Table 4). At 24 months post-transplantation, the triglyceride concentration remained higher in the TEP group. Absolute lymphocyte counts and hemoglobin concentration did not differ significantly over time between TEP and TMP groups in stratum A and B (Supplemental Figures 3 and 4).

**Table 4:** Laboratory values of special interest

Laboratory Measurement	TEP Group	TMP Group	Mean Difference (95% CI)
Proteinuria at 12 mo, mg/g	196 (114–359)	134 (90–246)	53 (20 to 80)
LDL cholesterol at 12 mo, mg/dl	93 (68–120)	81 (62–108)	8 (0 to 17)
Triglycerides at 12 mo, mg/dl	178 (133–253)	150 (109–198)	37 (19 to 52)
Proteinuria at 24 mo, mg/g	171 (110–318)	164 (93–308)	10 (−33 to 44)
LDL cholesterol at 24 mo, mg/dl	87 (65–113)	85 (64–100)	6 (−2 to 15)
Triglycerides at 24 mo, mg/dl	168 (123–216)	151 (108–190)	15 (−2 to 31)

All laboratory measurements are presented as median (25th percentile–75th percentile). CI, confidence interval; TEP, tacrolimus, everolimus, and prednisolone; TMP, tacrolimus, mycophenolate mofetil, and prednisolone.

## Discussion

The OPTIMIZE trial studied the effect of low-dose tacrolimus with everolimus and prednisolone versus standard-dose tacrolimus with MMF and prednisolone in 379 *de novo* older kidney transplant recipients. The trial showed no significant difference between these two regimens in reaching the primary end point of 2-year survival with graft function above a predefined threshold. In addition to the primary end point, the two treatment groups also did not differ regarding kidney function and patient and graft survival as individual outcomes. Moreover, no differences were observed in the incidence of allograft rejection or infectious complications.

When designing the trial, we assumed, based on an earlier analysis of the Dutch Transplant registry, that the proportion of participants with successful transplantation (survival with acceptable graft function) at 2 years after transplantation would be 48% in the control group.^1,10^ In the OPTIMIZE trial, the observed percentage of participants with successful transplantation in the control arm was 57%, reflecting a 10% improvement in transplant outcome in these participants over the past decade. This was true both for recipients of kidneys from older deceased donors in stratum A and for stratum B recipients who received a living donor kidney or a kidney from a deceased donor <65 years. In the intervention group, the percentage of successful transplantation was 50%. Thus, our hypothesis that the TEP regimen would improve success rates at 2 years after transplantation was not confirmed.

Regarding the secondary outcomes—patient survival, death-censored graft survival, and the prespecified eGFR threshold—most participants had favorable outcomes, with no significant differences between the treatment groups. Similarly, no differences were observed within the strata.

The primary end point, successful transplantation, excluded tBPAR, as previous studies have shown that the risk of acute rejection is lower in older recipients, especially in those receiving a kidney from a younger donor (<65 years) or from an older (≥65 years) donation after brain death donor.^1,12^ The incidence of tBPAR was indeed only 11% in the TEP group and 8% in the TMP group, consistent with expectations for this older population, and was not significantly different between groups. Further in-depth analysis of the OPTIMIZE study data is warranted to evaluate the effect of these rejections on short-term and long-term outcomes.

Regarding kidney function, we hypothesized that a lower CNI exposure would improve kidney function. However, eGFR values were favorable at 2 years after transplantation in both strata and did not differ significantly between treatment groups. In fact, eGFR improved over the 2 years of follow-up in both TEP and TMP groups. Similar to our findings, the TRANSFORM trial previously found no difference in kidney function between standard- and low-exposure tacrolimus arms.^8,9^ In contrast to the TRANSFORM study, OPTIMIZE included older recipients of mostly older potentially CNI-sensitive kidneys, including kidneys from death by circulatory death donors. Unlike TRANSFORM, where tacrolimus levels overlapped across study arms, OPTIMIZE achieved clear separation between low-dose tacrolimus and standard-dose tacrolimus throughout the entire study period. Therefore, inadequate separation of tacrolimus exposure cannot explain the lack of eGFR difference.

Previously, the ALLEGRO trial did not find a difference in eGFR at 2 years after transplantation between kidney transplant recipients targeted to lower (3–5 ng/ml) versus higher (6–8 ng/ml) target tacrolimus trough levels.^13^ By contrast, the BENEFIT and BENEFIT-EXT trials, which compared CNI-free belatacept regimens with ciclosporin, demonstrated significantly improved eGFR after 7 years.^14,15^ Possibly, complete elimination of CNIs is necessary to effectively reduce CNI-related nephrotoxicity. However, the SENATOR study in which 207 kidney transplant recipients participating in Eurotransplant Senior Program were randomized and switched from ciclosporine to everolimus at week 7 after transplantation also did not find a significant difference in eGFR at 6 months after transplantation.^16^ Alternatively, the combination of tacrolimus with everolimus may enhance nephrotoxicity by increasing tissue CNI levels and thus negating the beneficial effect of lower CNI dosing.^17,18^ Finally, our 2 year follow-up might have been too short to detect the effect of a reduction in CNI nephrotoxicity on kidney function.

Regarding the adverse events, we observed no significant differences in the incidence of infections between the two treatment groups. This contrasts with previous studies, including TRANSFORM, where infection rates, particularly viral infections, were lower with everolimus-based regimens.^8,9^ The larger TRANSFORM trial included 2037 participants, whereas OPTIMIZE included 379; thus, the discrepancy may reflect power limitations. However, OPTIMIZE showed no trend toward any difference in infection rates between the study groups. The relatively low MMF dose in OPTIMIZE (500 mg twice daily versus 750–1000 mg in TRANSFORM) may have contributed to similar infection rates in both study arms. Importantly, the OPTIMIZE trial was performed during the COVID-19 pandemic in a population at a high risk of infection. Of note, we previously reported enhanced vaccination response TEP compared with the TMP-treated kidney transplant recipients,^19^ yet in this study, no difference was observed in COVID-19 incidence nor COVID-19 outcome between TEP and TMP groups. Ongoing in-depth analyses of infections and immunosenescence in OPTIMIZE will hopefully provide more insight into the differential effect of both regimes on the immune response against viruses. Regarding other adverse events, there was a tendency toward fewer malignancies favoring the TEP group, but this difference was only found in nonskin malignancies. However, these findings should be interpreted with caution because the number of malignancies in our study was low, and immunosuppression-related malignancies are expected to occur after longer duration of treatment.

As expected, adverse events reflected drug-specific profiles, with significantly more edema or other signs of hypervolemia but a trend toward fewer neurologic side effects in TEP. Importantly, there was no significant difference in thromboembolic events, despite previous reports of a prothrombotic state in everolimus-treated patients.^20^ At 12 months levels of proteinuria, LDL cholesterol and triglycerides were higher in the TEP group, without persistence of all these differences at 24 months. These observed AEs and laboratory measurements are in line with previous observations in other studies, including TRANSFORM.^8,9^

Our study has some limitations. First, it was not powered to detect a benefit of the TEP regimen over TMP smaller than 14% for the primary end point as smaller differences were deemed clinically irrelevant at trial design. Second, the trial was open label, which is difficult to avoid since immunosuppressants must be titrated to trough levels.

Nevertheless, OPTIMIZE is one of the first randomized trials aiming to establish tailored immunosuppressive regimens specifically for older kidney transplant recipients. To the best of our knowledge, the only other randomized trial specifically investigating immunosuppression in older recipients compared delayed tacrolimus with basiliximab and MMF with early steroid discontinuation or standard tacrolimus with MMF and steroids.^21^ Interestingly, this trial did not show a benefit of delayed tacrolimus introduction in preserving kidney function of preventing delayed graft function. Overall, our trial demonstrated favorable 2-year transplant outcome of *de novo* older transplant recipients with a low drop-out rate. These findings again demonstrate that transplantation is a good and safe option for elderly patients with kidney failure. In subsequent analyses, we will address other important issues such as health-related quality of life and frailty.

In conclusion, a regimen of everolimus with low-dose tacrolimus did not improve rates of successful transplantation compared with a regimen consisting of MMF with standard-dose tacrolimus in older kidney transplant recipients. Similarly, kidney function and infection rates did not differ. However, both regimens appear safe and effective, offering therapeutic alternatives for the growing population of older kidney transplant recipients.

## Supplementary Material

## Data Availability

Original data generated for the study will be made available upon reasonable request to the corresponding author. Data Type: Clinical Trial Data. Reason for Restricted Access: Study data are available at request from the principal investigators of the study.

## Declarative Statements

This study includes clinical experimentation and received Institutional Review Board or Ethics Committee approval. All patients provided written informed consent. This study includes clinical experimentation and complies with the Declaration of Helsinki. The clinical and research activities being reported are consistent with the Principles of the Declaration of Istanbul as outlined in the Declaration of Istanbul on Organ Trafficking and Transplant Tourism.

## Supplemental Material

Supplemental Table 1. Inclusion and exclusion criteria for the OPTIMIZE study.

Supplemental Table 2. Participants with tacrolimus trough levels below, on, and above target at the study visits in the TEP and TMP groups.

Supplemental Table 3. Reasons for discontinuation of study medication for more than 21 days.

Supplemental Table 4. Serious adverse events.

Supplemental Figure 1. OPTIMIZE study design.

Supplemental Figure 2. MMF dosages in the TMP group according to stratum: (A) stratum A and (B) stratum B.

Supplemental Figure 3. Absolute lymphocyte counts (10E9/L; mean and 95% CI) in TEP and TMP groups in stratum A and B.

Supplemental Figure 4. Hemoglobulin concentration (mg/dl; mean and 95% CI) in TEP and TMP groups in stratum A and stratum B.

Study Protocol
