# Supplementary material for: Immunosuppression in Older Kidney Transplant Recipients: A Randomized Controlled Trial
Source: J Am Soc Nephrol. 2025 Nov 7;37(4):814–24. doi: 10.1681/ASN.0000000924 (PMC13065175; doi:10.1681/ASN.0000000924)
Supplement: Supplementary file 1 [file jasn-37-814-s001.pdf]

ASN American Society of Nephrology
JASN Journal of the American Society of Nephrology
KIDNEY360 Accessing Our World From Every Angle
CJASN Clinical Journal of the American Society of Nephrology

ASN Journal Disclosure Form

As per ASN journal policy, I have disclosed any financial relationships or commitments I have held in the past 36 months as included below. I have listed my Current Employer below to indicate there is a relationship requiring disclosure. If no relationship exists, my Current Employer is not listed.

F. Bemelman reports the following:
Employer: AMC; Consultancy: Astellas Pharma, HANSA; Advisory or Leadership Role: Astellas; Hansa; and Speakers Bureau: Hansa.

I understand that the information above will be published within the journal article, if accepted, and that failure to comply and/or to accurately and completely report the potential financial conflicts of interest could lead to the following: 1) Prior to publication, article rejection, or 2) Post-publication, sanctions ranging from, but not limited to, issuing a correction, reporting the inaccurate information to the authors' institution, banning authors from submitting work to ASN journals for varying lengths of time, and/or retraction of the published work.

Name: Frederike J. Bemelman
Manuscript ID: JASN-2025-001287
Manuscript Title: Results of The OPTIMIZE Study; A Randomized Clinical Trial in Older Kidney Transplant Recipients
Date of Completion: October 4, 2025
Disclosure Updated Date: October 4, 2025

ASN American Society of Nephrology | JASN Journal of the American Society of Nephrology | KIDNEY360 Accessing Our World From Every Angle | CJASN Clinical Journal of the American Society of Nephrology

ASN Journal Disclosure Form

As per ASN journal policy, I have disclosed any financial relationships or commitments I have held in the past 36 months as included below. I have listed my Current Employer below to indicate there is a relationship requiring disclosure. If no relationship exists, my Current Employer is not listed.

S. Berger reports the following:
Employer: UMCG; Research Funding: Chiesi; Novartis; Advisory or Leadership Role: Supervisory Board Dutch Transplant Foundation; and Speakers Bureau: Astellas.

I understand that the information above will be published within the journal article, if accepted, and that failure to comply and/or to accurately and completely report the potential financial conflicts of interest could lead to the following: 1) Prior to publication, article rejection, or 2) Post-publication, sanctions ranging from, but not limited to, issuing a correction, reporting the inaccurate information to the authors' institution, banning authors from submitting work to ASN journals for varying lengths of time, and/or retraction of the published work.

Name: Stefan P. Berger
Manuscript ID: JASN-2025-001287
Manuscript Title: esults of The OPTIMIZE Study; A Randomized Clinical Trial in Older Kidney Transplant Recipients
Date of Completion: October 3, 2025
Disclosure Updated Date: October 3, 2025

ASN American Society of Nephrology | JASN Journal of the American Society of Nephrology | KIDNEY360 Accessing Our World From Every Angle | CJASN Clinical Journal of the American Society of Nephrology

ASN Journal Disclosure Form

As per ASN journal policy, I have disclosed any financial relationships or commitments I have held in the past 36 months as included below. I have listed my Current Employer below to indicate there is a relationship requiring disclosure. If no relationship exists, my Current Employer is not listed.

M. Betjes reports the following:
Employer: Erasmus Medical Center; and Research Funding: Chiesi Pharmaceuticals; Astellas.

I understand that the information above will be published within the journal article, if accepted, and that failure to comply and/or to accurately and completely report the potential financial conflicts of interest could lead to the following: 1) Prior to publication, article rejection, or 2) Post-publication, sanctions ranging from, but not limited to, issuing a correction, reporting the inaccurate information to the authors' institution, banning authors from submitting work to ASN journals for varying lengths of time, and/or retraction of the published work.

Name: Michiel G.H. Betjes
Manuscript ID: JASN-2025-001287
Manuscript Title: LBCT: Results of The OPTIMIZE Study; A Randomized Clinical Trial in Older Kidney Transplant Recipients
Date of Completion: October 2, 2025
Disclosure Updated Date: October 2, 2025

ASN American Society of Nephrology | JASN Journal of the American Society of Nephrology | KIDNEY360 Accessing Our World From Every Angle | CJASN Clinical Journal of the American Society of Nephrology

ASN Journal Disclosure Form

As per ASN journal policy, I have disclosed any financial relationships or commitments I have held in the past 36 months as included below. I have listed my Current Employer below to indicate there is a relationship requiring disclosure. If no relationship exists, my Current Employer is not listed.

S. de Boer reports the following:
Employer: Univerisity Medical Centre Groningen

I understand that the information above will be published within the journal article, if accepted, and that failure to comply and/or to accurately and completely report the potential financial conflicts of interest could lead to the following: 1) Prior to publication, article rejection, or 2) Post-publication, sanctions ranging from, but not limited to, issuing a correction, reporting the inaccurate information to the authors' institution, banning authors from submitting work to ASN journals for varying lengths of time, and/or retraction of the published work.

Name: Silke de Boer
Manuscript ID: JASN-2025-001287
Manuscript Title: Results of The OPTIMIZE Study; A Randomized Clinical Trial in Older Kidney Transplant Recipients
Date of Completion: September 29, 2025
Disclosure Updated Date: September 29, 2025

ASN American Society of Nephrology | JASN Journal of the American Society of Nephrology | KIDNEY360 Accessing Our World From Every Angle | CJASN Clinical Journal of the American Society of Nephrology

ASN Journal Disclosure Form

As per ASN journal policy, I have disclosed any financial relationships or commitments I have held in the past 36 months as included below. I have listed my Current Employer below to indicate there is a relationship requiring disclosure. If no relationship exists, my Current Employer is not listed.

A. De Vries reports the following:
Employer: Leiden University Medical Center; and Consultancy: Hansa, Sanofi, Takeda;; all payments to employer.

I understand that the information above will be published within the journal article, if accepted, and that failure to comply and/or to accurately and completely report the potential financial conflicts of interest could lead to the following: 1) Prior to publication, article rejection, or 2) Post-publication, sanctions ranging from, but not limited to, issuing a correction, reporting the inaccurate information to the authors' institution, banning authors from submitting work to ASN journals for varying lengths of time, and/or retraction of the published work.

Name: Aiko P.J. De Vries
Manuscript ID: JASN-2025-001287
Manuscript Title: Optimize trial
Date of Completion: October 3, 2025
Disclosure Updated Date: October 3, 2025

ASN
American Society of Nephrology
JASN
Journal of the American Society of Nephrology
KIDNEY360
Accessing Our World From Every Angle
CJASN
Clinical Journal of the American Society of Nephrology

ASN Journal Disclosure Form

As per ASN journal policy, I have disclosed any financial relationships or commitments I have held in the past 36 months as included below. I have listed my Current Employer below to indicate there is a relationship requiring disclosure. If no relationship exists, my Current Employer is not listed.

D. Hesselink reports the following:
Employer: Erasmus MC; Consultancy: Astellas Pharma, Chiesi Pharma; Research Funding: Astellas Pharma, Chiesi Pharma; Honoraria: Astellas Pharma, Chiesi Pharmaceuticals; and Speakers Bureau: Astellas Pharma, Chiesi Pharma.

I understand that the information above will be published within the journal article, if accepted, and that failure to comply and/or to accurately and completely report the potential financial conflicts of interest could lead to the following: 1) Prior to publication, article rejection, or 2) Post-publication, sanctions ranging from, but not limited to, issuing a correction, reporting the inaccurate information to the authors' institution, banning authors from submitting work to ASN journals for varying lengths of time, and/or retraction of the published work.

Name: Dennis Alexander Hesselink
Manuscript ID: JASN-2025-001287
Manuscript Title: LBCT: Results of The OPTIMIZE Study; A Randomized Clinical Trial in Older Kidney Transplant Recipients
Date of Completion: September 30, 2025
Disclosure Updated Date: September 30, 2025

ASN American Society of Nephrology | JASN Journal of the American Society of Nephrology | KIDNEY360 Accessing Our World From Every Angle | CJASN Clinical Journal of the American Society of Nephrology

ASN Journal Disclosure Form

As per ASN journal policy, I have disclosed any financial relationships or commitments I have held in the past 36 months as included below. I have listed my Current Employer below to indicate there is a relationship requiring disclosure. If no relationship exists, my Current Employer is not listed.

L. Hilbrands reports the following:
Employer: Radboud University Medical Centre, Nijmegen, The Netherlands

I understand that the information above will be published within the journal article, if accepted, and that failure to comply and/or to accurately and completely report the potential financial conflicts of interest could lead to the following: 1) Prior to publication, article rejection, or 2) Post-publication, sanctions ranging from, but not limited to, issuing a correction, reporting the inaccurate information to the authors' institution, banning authors from submitting work to ASN journals for varying lengths of time, and/or retraction of the published work.

Name: Luuk Hilbrands
Manuscript ID: JASN-2025-001287
Manuscript Title: LBCT: Results of The OPTIMIZE Study; A Randomized Clinical Trial in Older Kidney Transplant Recipients
Date of Completion: September 30, 2025
Disclosure Updated Date: September 30, 2025

ASN American Society of Nephrology | JASN Journal of the American Society of Nephrology | KIDNEY360 Accessing Our World From Every Angle | CJASN Clinical Journal of the American Society of Nephrology

ASN Journal Disclosure Form

As per ASN journal policy, I have disclosed any financial relationships or commitments I have held in the past 36 months as included below. I have listed my Current Employer below to indicate there is a relationship requiring disclosure. If no relationship exists, my Current Employer is not listed.

M. Hilhorst reports the following:
Employer: Amsterdam UMC; Research Funding: Amsterdam UMC received funding for M.L. Hilhorst from the Dutch Kidney Foundation; and Speakers Bureau: Otsuka Pharma ; Astrazeneca.

I understand that the information above will be published within the journal article, if accepted, and that failure to comply and/or to accurately and completely report the potential financial conflicts of interest could lead to the following: 1) Prior to publication, article rejection, or 2) Post-publication, sanctions ranging from, but not limited to, issuing a correction, reporting the inaccurate information to the authors' institution, banning authors from submitting work to ASN journals for varying lengths of time, and/or retraction of the published work.

Name: Marc Hilhorst
Manuscript ID: JASN-2025-001287
Manuscript Title: LBCT: Results of The OPTIMIZE Study; A Randomized Clinical Trial in Older Kidney Transplant Recipients
Date of Completion: September 30, 2025
Disclosure Updated Date: March 7, 2025

ASN American Society of Nephrology
JASN Journal of the American Society of Nephrology
KIDNEY360 Accessing Our World From Every Angle
CJASN Clinical Journal of the American Society of Nephrology

ASN Journal Disclosure Form

As per ASN journal policy, I have disclosed any financial relationships or commitments I have held in the past 36 months as included below. I have listed my Current Employer below to indicate there is a relationship requiring disclosure. If no relationship exists, my Current Employer is not listed.

J. Jonker has nothing to disclose.

I understand that the information above will be published within the journal article, if accepted, and that failure to comply and/or to accurately and completely report the potential financial conflicts of interest could lead to the following: 1) Prior to publication, article rejection, or 2) Post-publication, sanctions ranging from, but not limited to, issuing a correction, reporting the inaccurate information to the authors' institution, banning authors from submitting work to ASN journals for varying lengths of time, and/or retraction of the published work.

Name: Jip Jonker
Manuscript ID: JASN-2025-001287
Manuscript Title: LBCT: Results of The OPTIMIZE Study; A Randomized Clinical Trial in Older Kidney Transplant Recipients
Date of Completion: October 1, 2025
Disclosure Updated Date: October 1, 2025

ASN American Society of Nephrology | JASN Journal of the American Society of Nephrology | KIDNEY360 Accessing Our World From Every Angle | CJASN Clinical Journal of the American Society of Nephrology

ASN Journal Disclosure Form

As per ASN journal policy, I have disclosed any financial relationships or commitments I have held in the past 36 months as included below. I have listed my Current Employer below to indicate there is a relationship requiring disclosure. If no relationship exists, my Current Employer is not listed.

D. Kuypers reports the following:
Employer: University Hospitals Leuven; Consultancy: Astellas Company, HANSA, GSK, Sangamo-Tx, Olaris Inc. and Takeda.; Honoraria: Astellas, HANSA and Takeda.; Advisory or Leadership Role: Associate editor Transplantation; Editorial board member Transplantation Reviews, Therapeutic Drug Monitoring, Current Clinical Pharmacology; and Speakers Bureau: Astellas, HANSA and Takeda.

I understand that the information above will be published within the journal article, if accepted, and that failure to comply and/or to accurately and completely report the potential financial conflicts of interest could lead to the following: 1) Prior to publication, article rejection, or 2) Post-publication, sanctions ranging from, but not limited to, issuing a correction, reporting the inaccurate information to the authors’ institution, banning authors from submitting work to ASN journals for varying lengths of time, and/or retraction of the published work.

Name: Dirk R. Kuypers
Manuscript ID: JASN-2025-001287
Manuscript Title: ("LBCT: Results of The OPTIMIZE Study; A Randomized Clinical Trial in Older Kidney Transplant Recipients"
Date of Completion: September 30, 2025
Disclosure Updated Date: September 30, 2025

ASN
American Society of Nephrology
JASN
Journal of the American Society of Nephrology
KIDNEY360
Accessing Our World From Every Angle
CJASN
Clinical Journal of the American Society of Nephrology

ASN Journal Disclosure Form

As per ASN journal policy, I have disclosed any financial relationships or commitments I have held in the past 36 months as included below. I have listed my Current Employer below to indicate there is a relationship requiring disclosure. If no relationship exists, my Current Employer is not listed.

J. Sanders reports the following:
Employer: University Medical Center Groningen; Research Funding: Chiesi, Novartis; and Other Interests or Relationships: Independent Data Safety Monitoring Board ALENTIS Therapeutics.

I understand that the information above will be published within the journal article, if accepted, and that failure to comply and/or to accurately and completely report the potential financial conflicts of interest could lead to the following: 1) Prior to publication, article rejection, or 2) Post-publication, sanctions ranging from, but not limited to, issuing a correction, reporting the inaccurate information to the authors' institution, banning authors from submitting work to ASN journals for varying lengths of time, and/or retraction of the published work.

Name: Jan-Stephan Sanders
Manuscript ID: JASN-2025-001287
Manuscript Title: LBCT: Results of The OPTIMIZE Study; A Randomized Clinical Trial in Older Kidney Transplant Recipients
Date of Completion: September 30, 2025
Disclosure Updated Date: September 30, 2025

American Society of Nephrology | JASN Journal of the American Society of Nephrology | KIDNEY360 Accessing Our World From Every Angle | CJASN Clinical Journal of the American Society of Nephrology

ASN Journal Disclosure Form

As per ASN journal policy, I have disclosed any financial relationships or commitments I have held in the past 36 months as included below. I have listed my Current Employer below to indicate there is a relationship requiring disclosure. If no relationship exists, my Current Employer is not listed.

A. Van Zuilen reports the following:
Employer: UMC Utrecht; and Research Funding: Chiessi pharmaceuticals.

I understand that the information above will be published within the journal article, if accepted, and that failure to comply and/or to accurately and completely report the potential financial conflicts of interest could lead to the following: 1) Prior to publication, article rejection, or 2) Post-publication, sanctions ranging from, but not limited to, issuing a correction, reporting the inaccurate information to the authors' institution, banning authors from submitting work to ASN journals for varying lengths of time, and/or retraction of the published work.

Name: Arjan D. Van Zuilen
Manuscript ID: JASN-2025-001287
Manuscript Title: LBCT: Results of The OPTIMIZE Study; A Randomized Clinical Trial in Older Kidney Transplant Recipients
Date of Completion: September 30, 2025
Disclosure Updated Date: September 30, 2025

ASN
American Society of Nephrology
JASN
Journal of the American Society of Nephrology
KIDNEY360
Accessing Our World From Every Angle
CJASN
Clinical Journal of the American Society of Nephrology

ASN Journal Disclosure Form

As per ASN journal policy, I have disclosed any financial relationships or commitments I have held in the past 36 months as included below. I have listed my Current Employer below to indicate there is a relationship requiring disclosure. If no relationship exists, my Current Employer is not listed.

P. Vart reports the following:
Employer: University Medical Center Groningen; Ownership Interest: Apple, Tesla, MicroSoft, Nio, Airbus, Boeing; Research Funding: AstraZeneca; and Advisory or Leadership Role: Editor Nephrology Dialysis Transplantation (unpaid);.

I understand that the information above will be published within the journal article, if accepted, and that failure to comply and/or to accurately and completely report the potential financial conflicts of interest could lead to the following: 1) Prior to publication, article rejection, or 2) Post-publication, sanctions ranging from, but not limited to, issuing a correction, reporting the inaccurate information to the authors' institution, banning authors from submitting work to ASN journals for varying lengths of time, and/or retraction of the published work.

Name: Priya Vart
Manuscript ID: JASN-2025-001287
Manuscript Title: LBCT: Results of The OPTIMIZE Study; A Randomized Clinical Trial in Older Kidney Transplant Recipients
Date of Completion: October 4, 2025
Disclosure Updated Date: October 4, 2025
