# Supplementary material for: Immunosuppression in Older Kidney Transplant Recipients: A Randomized Controlled Trial
Source: J Am Soc Nephrol. 2025 Nov 7;37(4):814–24. doi: 10.1681/ASN.0000000924 (PMC13065175; doi:10.1681/ASN.0000000924)
Supplement: Supplementary file 2 [file jasn-37-814-s002.pdf]

Supplemental Material

Supplemental Table 1: Inclusion and exclusion criteria for the OPTIMIZE study

Supplemental Table 2. Participants with tacrolimus trough levels below, on, and above target at the study visits in the TEP and TMP groups

Supplemental Table 3. Reasons for discontinuation of study medication for more than 21 days

Supplemental Table 4. Serious Adverse Events

Supplemental Figure 1. OPTIMIZE study design

Supplemental Figure 2. MMF dosages in the TMP-group according to Stratum: A = Stratum A, B = Stratum B.

Supplemental Figure 3. Absolute lymphocyte counts (10E9/L; mean and 95% CI) in TEP and TMP groups in stratum A and B.

Supplemental Figure 4. Hemoglobulin concentration (mg/dL) (mean and 95% CI) in TEP and TMP groups in Stratum A and Stratum

Study Protocol

Supplemental Table 1: Inclusion and exclusion criteria for the OPTIMIZE study

**Inclusion criteria**	**Exclusion criteria (for both strata)**
1. Written informed consent must be obtained before any assessment is performed	1. Subject is a multi-organ transplant recipient
2. Male or female subject ≥65 years old	2. Recipient of bloodgroup ABO incompatible allograft or CDC cross-match positive transplant
3. Subject randomized within 24 hours of completion of transplant surgery	3. Subject at high immunological risk for rejection as determined by local practice for assessment of anti-donor reactivity
4. Stratum A: Recipient of a primary (or secondary, if first graft is not lost due to immunological reasons) renal transplant from a deceased donor aged 65 years or older	4. Recipient of a kidney with a cold ischemia time (CIT) >24 hours
5. Stratum B: Recipient of a primary (or secondary, if first graft is not lost due to immunological reasons) renal transplant from a deceased donor aged below 65 years or a living donor of any age	5. Recipients of a kidney from an HLA-identical related living donor
	6. Known intolerability for one or more of the study drugs
	7. Subject who is HIV positive
	8. HBsAg and/or a HCV positive subject with evidence of elevated liver function tests (ALT/AST levels ≥2.5 times ULN). Viral serology results obtained within 6 months prior to randomization are acceptable
	9. Recipient of a kidney from a donor who tests positive for human immunodeficiency virus (HIV), hepatitis B surface antigen (HBsAg) or anti-hepatitis C virus (HCV)
	10. Subject with severe systemic infections, current or within the two weeks prior to randomization
	11. Subject with severe restrictive or obstructive pulmonary disorders
	12. Subject with severe hypercholesterolemia or hypertriglyceridemia that cannot be controlled
	13. Subject with white blood cell (WBC) count ≤ 2,000/mm^3 or with platelet count ≤ 50,000/mm^3

Supplemental Table 2. Participants with tacrolimus trough levels below, on, and above target at the study visits in the TEP and TMP groups

	**TEP group**			**TMP group**		
**Visit**	**Below target**	**On target**	**Above target**	**Below target**	**On target**	**Above target**
**Day 7**	41 (23.2%)	57 (32.2%)	79 (44.6%)	81 (44%)	69 (37.5%)	34 (18.5%)
**1 Month**	22 (13.5%)	65 (39.9%)	76 (46.6%)	40 (21.7%)	94 (51.1%)	50 (27.2%)
**3 Months**	18 (12.8%)	55 (39%)	68 (48.2%)	43 (26.2%)	86 (52.4%)	35 (21.3%)
**6 Months**	2 (1.7%)	58 (50.4%)	55 (47.8%)	12 (9.8%)	80 (65%)	31 (25.2%)
**9 Months**	3 (3%)	64 (64%)	33 (33%)	18 (15.5%)	70 (60.3%)	28 (24.1%)
**12 Months**	2 (2.1%)	68 (72.3%)	24 (25.5%)	23 (21.3%)	61 (56.5%)	24 (22.2%)
**18 Months**	2 (2.5%)	58 (72.5%)	20 (25%)	24 (25.3%)	53 (55.8%)	18 (18.9%)
**24 Months**	4 (5.1%)	59 (75.6%)	15 (19.2%)	19 (21.3%)	56 (62.9%)	14 (15.7%)

Supplemental Table 3. Reasons for discontinuation of study medication for more than 21 days

	TEP	TMP
Due to adverse events, n	64	62
Due to decline of kidney function, n	1	0
Due to suspected acute rejection, n	3	2
Due to other reason, n	7	9

Supplemental Table 4. Serious Adverse Events

Serious Adverse Event category	TEP	TMP	Relative Risk (95% CI)	P value
Immune system disorders	16 (8.6%)	14 (7.3%)	1.17 (0.59 to 2.34)	0.65
Infections and infestations	73 (39%)	78 (40.6%)	0.96 (0.75 to 1.23)	0.75
Cardiac disorders	19 (10.2%)	17 (8.9%)	1.15 (0.62 to 2.14)	0.66
Metabolism and nutrition disorders	12 (6.4%)	11 (5.7%)	1.12 (0.51 to 2.48)	0.78
Injury, poisoning and procedural complications	19 (10.2%)	14 (7.3%)	1.39 (0.72 to 2.70)	0.32
Surgical and medical procedures	14 (7.5%)	15 (7.8%)	0.96 (0.48 to 1.93)	0.91
Investigations	9 (4.8%)	12 (6.2%)	0.77 (0.33 to 1.78)	0.54
Renal and urinary disorders	29 (15.5%)	17 (8.9%)	1.75 (1.00 to 3.08)	0.05
General disorders and administration site conditions	16 (8.6%)	18 (9.4%)	0.91 (0.48 to 1.74)	0.78
Gastrointestinal disorders	19 (10.2%)	20 (10.4%)	0.98 (0.54 to 1.77)	0.93
Psychiatric disorders	5 (2.7%)	7 (3.6%)	0.73 (0.24 to 2.27)	0.59
Respiratory, thoracic and mediastinal disorders	13 (7%)	11 (5.7%)	1.21 (0.56 to 2.64)	0.63
Vascular disorders	10 (5.3%)	7 (3.6%)	1.47 (0.57 to 3.77)	0.42
Blood and lymphatic system disorders	6 (3.2%)	3 (1.6%)	2.05 (0.52 to 8.09)	0.29
Musculoskeletal and connective tissue disorders	5 (2.7%)	3 (1.6%)	1.71 (0.41 to 7.06)	0.45
Neoplasms benign, malignant and unspecified (incl cysts and polyps)	4 (2.1%)	11 (5.7%)	0.37 (0.12 to 1.15)	0,07
Hepatobiliary disorders	3 (1.6%)	1 (0.5%)	3.08 (0.32 to 29.35)	0.30
Nervous system disorders	5 (2.7%)	6 (3.1%)	0.86 (0.27 to 2.76)	0.79
Endocrine disorders	1 (0.5%)	0 (0%)	Not applicable	0.31
Skin and subcutaneous tissue disorders	1 (0.5%)	1 (0.5%)	1.03 (0.06 to 16.30)	0.99
Reproductive system and breast disorders	0 (0%)	2 (1%)	Not applicable	0.16

Supplemental Figure 1

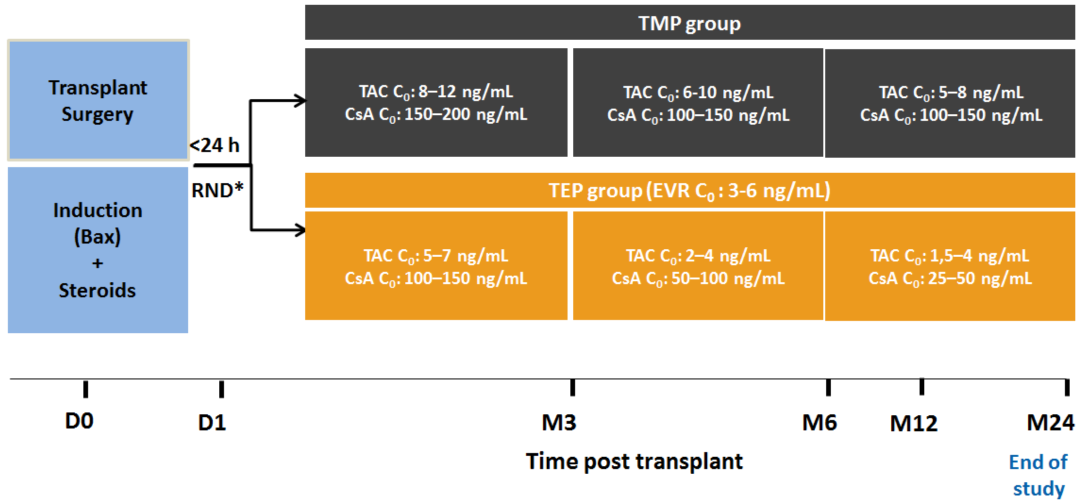

Supplemental Figure 1: OPTIMIZE study design
TMP = tacrolimus, mycophenolate mofetil, prednisolon, TAC = tacrolimus, CsA = cyclosporin A, BAX = basiliximab, RND = randomization, TEP = tacrolimus, everolimus, prednisolone, EVR = everolimus

Supplemental Figure 2

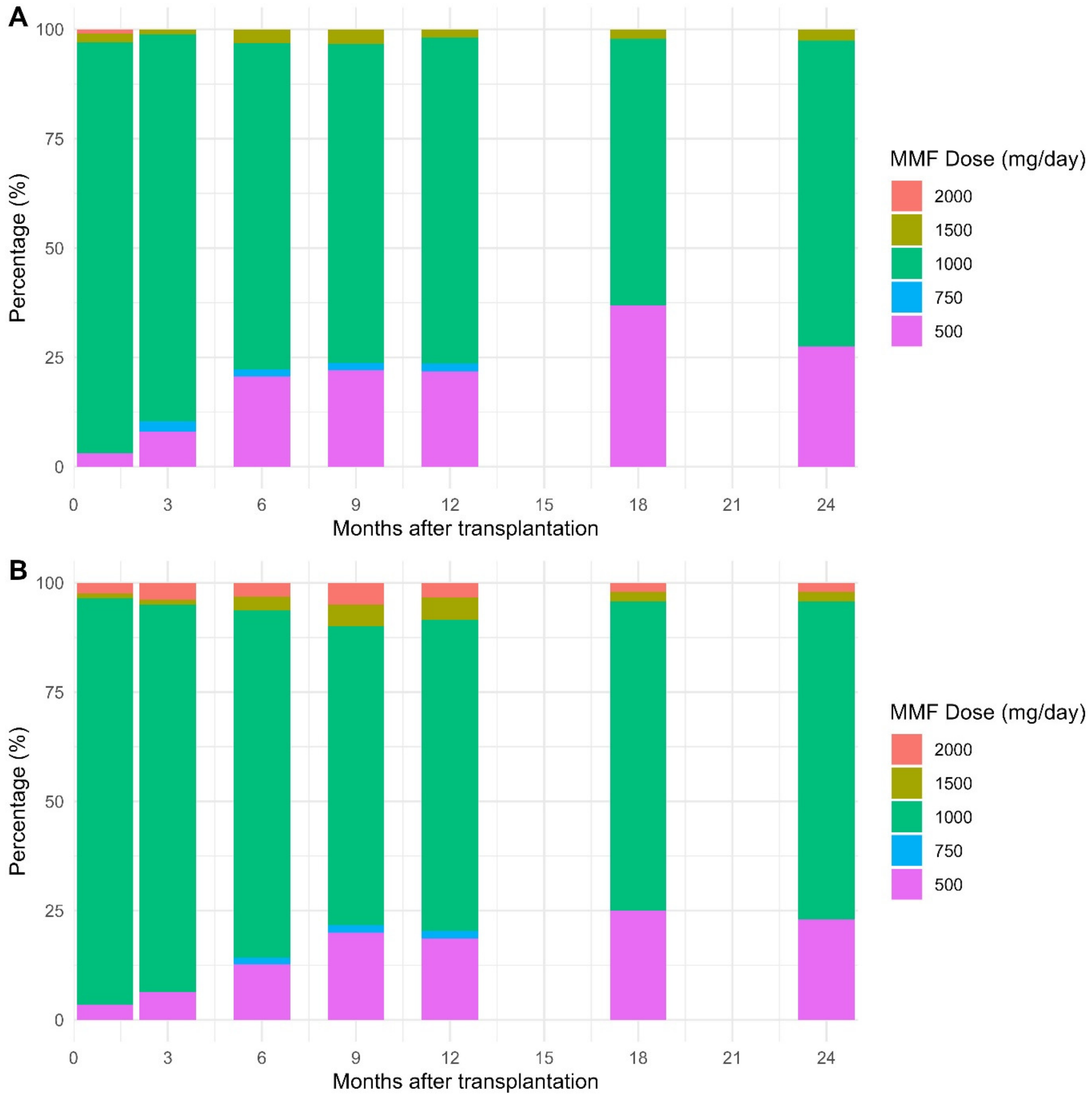

Supplemental Figure 2. MMF dosages in the TMP-group according to Stratum: A = Stratum A, B = Stratum B.

Supplemental Figure 3

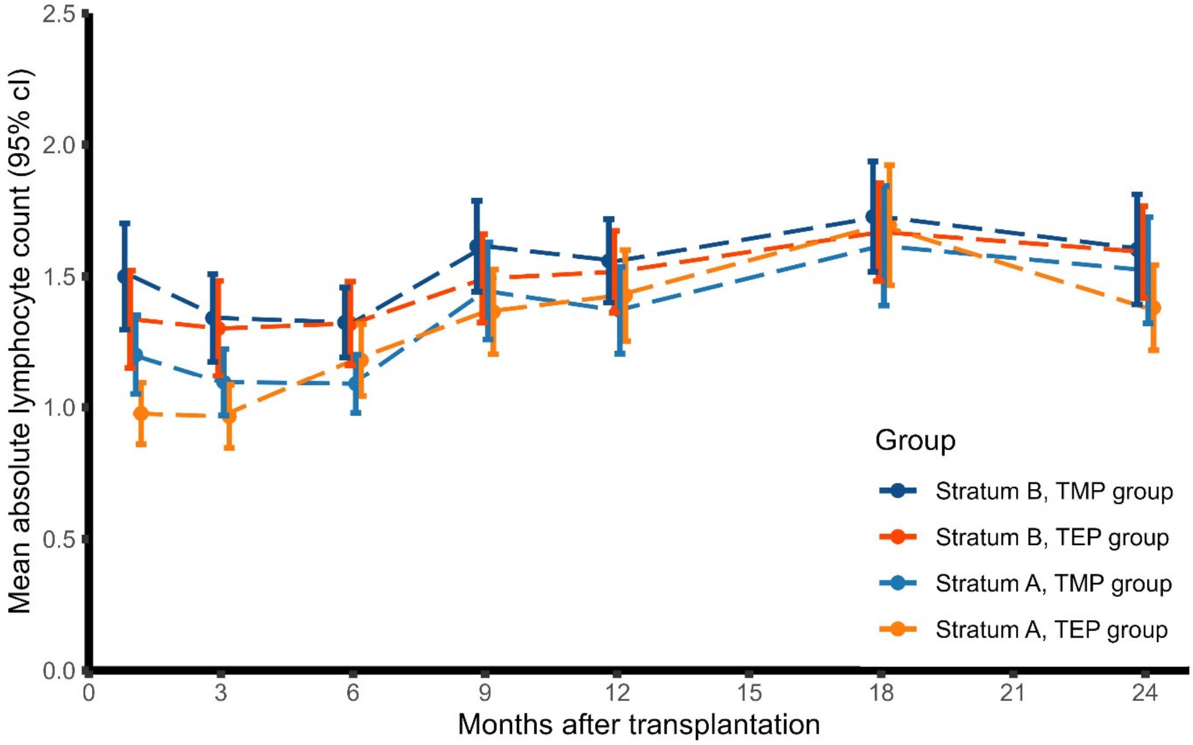

Supplemental Figure 3. Absolute lymphocyte counts (10E9/L; mean and 95% CI) in TEP and TMP groups in stratum A and B.

Supplemental Figure 4

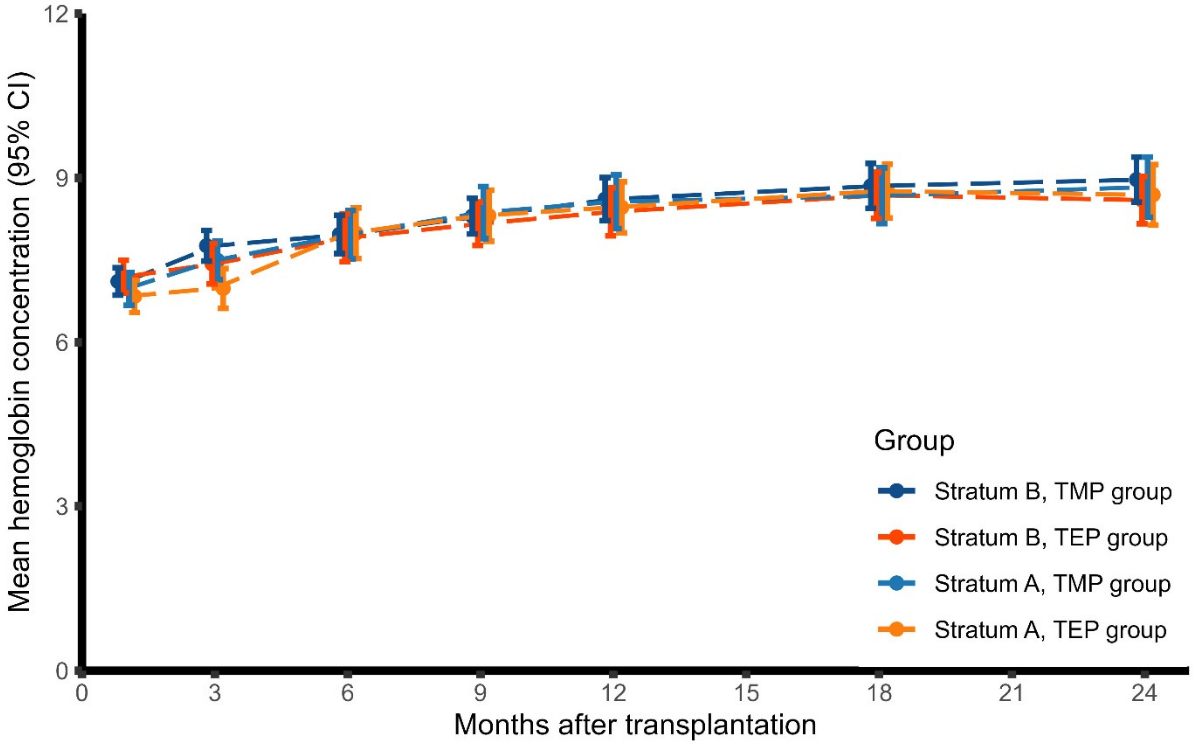

Supplemental Figure 4. Hemoglobulin concentration (mg/dL) (mean and 95% CI) in TEP and TMP groups in Stratum A and Stratum B.

OPen label multicenter randomized **T**rial comparing standard **IM**munosuppression with tacrolimus and mycophenolate mofetil with a low exposure tacrolimus regimen **I**n combination with everolimus in the novo renal transplantation in **E**lderly patients.

OPTIMIZE

PROTOCOL TITLE 'Open label multicenter randomized trial comparing standard immunosuppression with tacrolimus and mycophenolate mofetil with a low exposure tacrolimus regimen in combination with everolimus in the novo renal transplantation in elderly patients.'

**Protocol ID**	Open label multicenter randomized trial comparing standard immunosuppression with tacrolimus and mycophenolate mofetil with a low exposure tacrolimus regimen in combination with everolimus in the novo renal transplantation in elderly patients.
**Short title**	OPTIMIZE
**EudraCT number**	follows
**Version**	01
**Date**	4-10-2017
**project leader**	S.P. Berger<br>s.p.berger@umcg.nl
**Principal investigator(s)**	*J.S.F. Sanders, UMCG, D.A. Hesselink, Erasmus MC F.J. Bemelman, AMC, S.A. Nurmohamed, VUMC A.P.J. de Vries, LUMC, L.Hilbrands, Radboud UMC A.D. van Zuilen UMCU, D. Kuypers, UZ Leuven*<br>*T. van Gelder, Erasmus MC, internist-nephrologist S de Rooij, UMCG, geriatrician, M. Betjes, Erasmus MC, internist-nephrologist, M. Postma, RUG, Pharmaco-economist, J.G.M. Burgerhof, UMCG, statistician*
**Sponsor (in Dutch: verrichter/opdrachtgever)**	S.P. Berger<br>s.p.berger@umcg.nl
**Subsidising party**	-
**Independent expert (s)**	Members of the DSMB:<br>*prof. dr. A.J. Hoitsma (chair), retired internist-nephrologist, and dr. E.F. de Maar, internist-nephrologists WZA, dr. A. Zwinderman, AMC, epidemiologist*

PROTOCOL SIGNATURE SHEET

**Name**	**Signature**	**Date**
**Sponsor or legal representative:**	S.P Berger, head of department of nephrology	
**Coordinating Investigator/Project leader/Principal Investigator**	S.P Berger, head of department of nephrology	

TABLE OF CONTENTS

1. INTRODUCTION AND RATIONALE 9
2. OBJECTIVES 12
3. STUDY DESIGN 13
4. STUDY POPULATION 16
4.1 Population (base) 16
4.2 Inclusion criteria 16
4.3 Exclusion criteria 16
4.4 Sample size calculation 17
5. TREATMENT OF SUBJECTS 17
5.1 Product requested treatment 16
5.2 Permitted dose adjustments and interruptions of study treatment 18
5.3 Concomitant treatment 20
5.4 Prohibited Treatment 21
5.5 Discontinuation of study treatment and discontinuation from study 21
5.6 Randomization 22
6. METHODS 23
6.1 Study procedures 23
6.2 Withdrawal of individual subjects 23
6.3 Premature termination of the study 23
7. SAFETY REPORTING 23
7.1 Section 10 WMO event 23
7.2 Adverse and serious adverse events 24
7.3 Suspected unexpected serious adverse reactions (SUSAR) 24
7.4 Annual Safety report 25
7.5 Follow-up of adverse events 25
7.6 Data Safety Monitoring Board 25
8. STATISTICAL ANALYSIS 26
9. ETHICAL CONSIDERATIONS 28
9.1 Regulation statement 28
9.2 Compensation for injury 27
10. ADMINISTRATIVE ASPECTS, MONITORING AND PUBLICATION 28
10.1 Monitoring and Quality Assurance 28
10.2 Amendments 29
10.3 Annual progress report 29
10.4 Temporary halt and (prematurely) end of study report 29
11. REFERENCES 29

LIST OF ABBREVIATIONS AND RELEVANT DEFINITIONS

ABR	ABR form, General Assessment and Registration form, is the application form that is required for submission to the accredited Ethics Committee (In Dutch, ABR = Algemene Beoordeling en Registratie)
AE	Adverse Event
AR	Adverse Reaction
CA	Competent Authority
CCMO	Central Committee on Research Involving Human Subjects; in Dutch: Centrale Commissie Mensgebonden Onderzoek
CV	Curriculum Vitae
DSMB	Data Safety Monitoring Board
EU	European Union
EudraCT	European drug regulatory affairs Clinical Trials
GCP	Good Clinical Practice
IB	Investigator’s Brochure
IC	Informed Consent
IMP	Investigational Medicinal Product
IMPD	Investigational Medicinal Product Dossier
METC	Medical research ethics committee (MREC); in Dutch: medisch ethische toetsing commissie (METC)
(S)AE	(Serious) Adverse Event
SPC	Summary of Product Characteristics (in Dutch: officiële productinfomatie IB1-tekst)
Sponsor	The sponsor is the party that commissions the organisation or performance of the research, for example a pharmaceutical company, academic hospital, scientific organisation or investigator. A party that provides funding for a study but does not commission it is not regarded as the sponsor, but referred to as a subsidising party.
SUSAR	Suspected Unexpected Serious Adverse Reaction
Wbp	Personal Data Protection Act (in Dutch: Wet Bescherming Persoonsgevens)
WMO	Medical Research Involving Human Subjects Act (in Dutch: Wet Medisch-wetenschappelijk Onderzoek met Mensen

PROTOCOL SYNOPSIS

Rationale

Elderly patients increasingly contribute to both the dialysis and transplant population. In 2015 more than 30% of the transplant patients in the Netherlands were above 65 years of age while more than 50% of the dialysis population is older than 65 years. Kidney transplantation has some important age-dependent characteristics. Older patients with increased frailty and co-morbidity clearly have different risk profiles when compared with younger patients. While graft loss in younger patients is largely due to loss of the kidney with the recipient needing an alternative form of renal function replacement (e.g. dialysis and/or re-transplantation), death censored graft loss is a relatively rare phenomenon in older patients. Increased rates of malignancy and infection-related mortality have been reported in older transplant recipients. On the other hand, it has become clear that the aging immune system renders elderly recipients less prone to rejection. Kidneys from elderly donors are preferentially allocated to older recipients. A recent analysis of the results of deceased donor kidney transplantation in the elderly showed that in these patients graft loss is dominated by patient loss. Poor renal function in these patients may be both related to chronic damage of the kidney prior to transplantation (due to older donor age), and to an increased susceptibility to the toxicity of the immune suppressant tacrolimus. Especially in elderly recipients receiving marginal grafts it is essential to shift the focus from prevention of rejection to a stronger focus on preservation of graft function and preventing over-immunosuppression.

In this study two immunosuppressive regimes will be tested; the standard therapy consisting of prednisolone, mycophenolate acid and tacrolimus once daily (Envarsus®), or the comparator in which mycophenolate acid will be replaced by everolimus combined with strongly reduced levels of tacrolimus once daily (Envarsus®). The hypothesis is that reduced CNI exposure will lead to improved allograft function, a reduced incidence of complications and improved quality of life. This study will consist of two parts: Part A: Elderly recipients (≥65 years) of kidneys from elderly deceased donors (≥65 years) within the Eurotransplant Senior Program

Part B: Elderly recipients (≥65 years) of kidneys from living donors (all ages) or deceased donors (<65 years). The primary endpoint will be successful transplantation defined as survival with a functioning allograft with a minimum estimated GFR of 30 ml/min in part A and 45 ml/min in part B, after 1 year.

The study will be performed by the Dutch transplant centers and the Dutch Kidney Patient Organisation (NVN) will participate. This study will form a starting point for future collaboration between the renal transplant groups of the university medical centers in The Netherlands, who never before have participated together in a prospective clinical trial. Furthermore, this study will provide important guidance for the treatment of the elderly renal transplant population.

Objective

1. To test the hypothesis that an age adapted immunosuppressive regimen targeted at reduced immunosuppression with low calcineurin inhibitor exposure will result in improved outcome in elderly recipients of A: kidneys from older deceased donors and B: Kidneys from living donors and younger deceased donors.
2. To evaluate the impact of transplantation and adapted immunosuppression on frailty and quality of life in older Dutch transplant recipients
3. To monitor the function of the aged immune system after transplantation and the effect of everolimus based immunosuppression on parameters of immunosenescence compared to standard tacrolimus based immunosuppression.
4. To establish a national platform for trials in kidney transplantation as a basis for future high impact clinical trials.
5. To identify immunologic parameters that may serve as biomarkers of immunosenescence for future clinical application.

Study design

Open label randomised national multicentre intervention trial comparing standard immunosuppression with tacrolimus and mycophenolate mofetil with a low exposure tacrolimus regimen in combination with everolimus.

The trial will consist of two parts:

Part A: Elderly recipients (≥65 years) of kidneys from elderly deceased donors (≥65 years) within the Eurotransplant Senior Program

Part B: Elderly recipients (≥65 years) of kidneys from living donors (all ages) or deceased donors (<65 years).

Study population

Part A: Elderly recipients (≥65 years) receiving a kidney transplant from elderly deceased donors (≥65 years) within the Eurotransplant Senior Program (both DCD and DBD donors)

Part B: Part B: Elderly recipients (≥65 years) receiving a kidney transplant from either a living donor (all ages) or a deceased donor (<65 years) within regular Eurotransplant allocation (ETKAS).

Intervention

Study patients will be randomized to the following regime in both part A and part B in a 1:1ratio.

Arm 1: Basiliximab (B) induction (20 mg iv on days 0 and 4), prednisolone taper to 5 mg at 3 months after transplantation, tacrolimus once daily (Envarsus®) with an initial target trough level

of 8-12 tapered to 5-8ug/l at 6 months after transplantation, mycophenolate mofetil at dose of 500 mg bd throughout the trial.

Arm 2: Basiliximab (B) induction (20 mg iv on days 0 and 4), prednisolone taper to 5 mg at 3 months after transplantation, tacrolimus once daily (Envarsus®) with initial target trough level of 5-7 tapered to 2-4 ug/l from 3 months, and 1.5-4 ug/l from 6 months after transplantation, everolimus (EVL) will be initiated at a starting dose of 1.5 mg bid with target trough level of 3-6 ug/l throughout the trial.

Arm 1

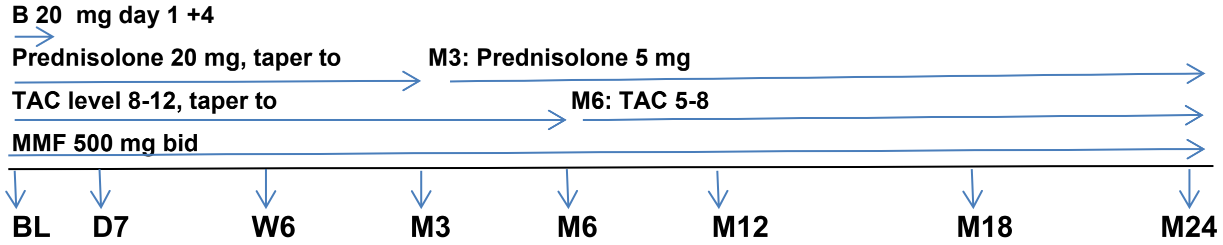

Arm 2

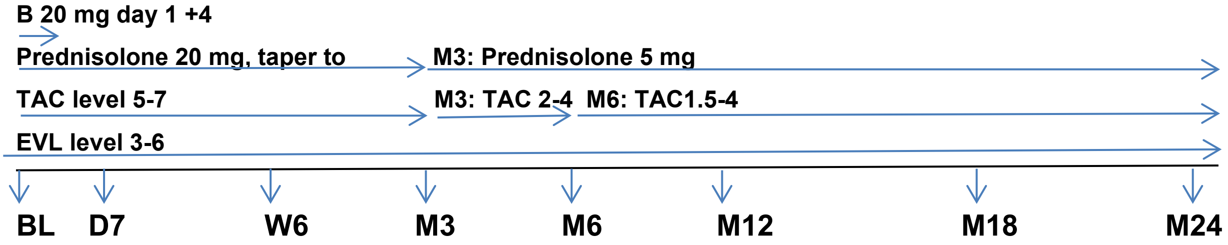

1. INTRODUCTION AND RATIONALE

Elderly patients increasingly contribute to both the dialysis and transplant population. In 2015 more than 30% of the transplant patients in the Netherlands were above 65 years of age while more than 50% of the dialysis population is older than 65 years. This shift towards older recipients is a major challenge for modern transplant medicine and all professionals involved in the care for the older patient with renal failure.

Kidney transplantation has some important age-dependent characteristics. Older patients with increased frailty and co-morbidity clearly have different risk profiles when compared with younger patients. These challenges do not only pertain to patient management after transplantation but also have important implications for decision making prior to the initiation of renal replacement therapy.

While graft loss in younger patients is largely due to loss of the kidney with the recipient needing an alternative form of renal function replacement (e.g. dialysis and/or re-transplantation), death censored graft loss is a relatively rare phenomenon in older patients. This is not only explained by the higher competing incidence of death in elderly patients but also by specific differences in

immunity and organ quality. Increased rates of malignancy and infection-related mortality have been reported in older transplant recipients[1 2]. On the other hand, it has become clear that the aging immune system renders elderly recipients less prone to rejection [3]. This decreased propensity for rejection is explained by senescence of adaptive immunity with decreasing telomere length of immune cells and a shift from a naïve T-cell repertoire towards terminally differentiated memory T-cells [4]. Additionally, a shift towards an increasing proportion of CD28 negative T-cells has been noted with important implications for rejection risk and possibly for the choice of immunosuppression in older recipients [5 6].

Not only recipient characteristics are important determinants in older transplant recipients. In the Netherlands, kidneys from elderly donors are preferentially allocated to older recipients. In a recent analysis of the results of deceased donor kidney transplantation in the elderly in the Netherlands the following was noted [7]: In elderly patients graft loss is dominated by patient loss. Death-censored graft loss later than 3 months after transplantation was a rare event in elderly recipients. However, patient death was a frequent event, with less than 50% of patients being alive at 5 years after transplantation. Additionally, we demonstrated that more than 50% of patients older than 65 years who received a graft within the Eurotransplant Senior Program (ESP) have an eGFR below 30 ml/min at 1 year after transplantation. Poor renal function in these patients may be both related to chronic damage of the kidney prior to transplantation (due to older donor age), and to an increased susceptibility to the toxicity of the calcineurin inhibitors (CNI, e.g. cyclosporine and tacrolimus), the cornerstone immunosuppressant. This increased susceptibility may be partially explained by altered pharmacokinetics in elderly recipients [8]. More importantly, a number of observations indicate that both transplanted and native older kidneys are inherently more susceptible to acute and chronic toxicity of these drugs [9 10] .

From the findings stated above it is obvious that older transplant recipients require specific considerations with an immunosuppressive approach that balances the reduced alloreactivity of the senescent immune system with the increased susceptibility to CNI toxicity of the aged allograft. If we wish to improve outcomes in older transplant recipients we will have to target mortality and poor renal function and thus shift from the traditional approach in which the prevention of acute rejection is the central goal.

Based on these considerations this study is based on the following assumptions:

1. Outcome in elderly transplant recipients is dominated by patient death and not by rejection or death censored graft loss.
2. Complications of immunosuppressive medication play an important role in increased mortality of elderly transplant recipients.
3. The use of organs from elderly donors in elderly recipients is associated with worse outcome due to increased susceptibility of the transplanted kidney to CNI toxicity

4. There is an unmet clinical need to select an age specific immunosuppressive regimen for the elderly transplant recipient.

Especially in elderly recipients receiving marginal grafts it is essential to shift the focus from prevention of rejection to a stronger focus on preservation of graft function and preventing over-immunosuppression. Elimination of CNIs is currently only possible with one registered regime based on the use of CTLA4-Ig, Belatacept. However, as Belatacept is dependent on blocking the CD80/86 CD28 pathway this drug may not be ideal in elderly recipients. As described above, immunosenescence is characterised by a loss of CD28+ cells and thus the drug may lack efficiency in this population. Additionally, Belatacept is administered by in-hospital monthly infusions. This may be an unacceptable burden for a patient population with reduced mobility, and with problematic vascular access (dialysis shunts). The use of the mTOR inhibitor (mTORi) everolimus in combination with reduced tacrolimus exposure instead of mycophenolate mofetil with standard tacrolimus exposure is an attractive alternative. Studies have indicated that reduced CNI exposure is possible in the combination with mTOR inhibitors [11 12]. This alternative combination was safe with similar rates of rejection when compared with standard treatment. The results of a multicenter trial comparing low dose tacrolimus and everolimus with standard dose tacrolimus and mycophenolic acid are expected in the course of this year [13]. Reduced tacrolimus exposure in combination with everolimus is especially attractive in elderly recipients as a low CNI dose is expected to lead to improved graft function specifically in the case of marginal grafts with pre-existing vascular damage. As rejection is almost exclusively seen early after transplantation in elderly recipients, further reduction of immunosuppression is expected to be safe at 6 months after transplantation and likely results in improvement of graft function, less cardiovascular side effects and a decrease in infectious complications.

This study will consist of two parts. Study A will specifically address older recipients receiving kidneys from older donors within the Eurotransplant Senior Program. As discussed above, although this program has resulted in a reduction of waiting times and competition on the waiting list, the results of ESP in terms of patient survival and kidney function are far from optimal. We hypothesize that reduced CNI exposure will lead to improved allograft function, a reduced incidence of complications and improved quality of life. The primary endpoint will be successful transplantation at 1 year after transplantation defined as survival with a functioning allograft with an estimated GFR above 30 ml/min. Study B will include elderly recipients receiving either a kidney from deceased donors younger than 65 years or a living donor kidney. The primary endpoint will be successful transplantation defined as survival with a functioning allograft with an estimated GFR above 45 ml/min.

Both parts will include kidney allograft recipients aged 65 years and older. This age limit is primarily chosen because current Eurotransplant allocation includes recipients and donors in the Eurotransplant Senior Program from an age of 65 and upwards.

Successful patient inclusion necessitates support of the Dutch transplant centers. The Dutch Kidney Transplantation programs are organized within the Dutch Kidney Advisory Board (Landelijk Overleg Niertransplantatie, LONT). This advisory board aims to coordinate and strengthen the efforts to improve the care for the transplant patients in the Netherlands and is a communication partner for the Dutch Transplant Foundation (NTS), the Dutch Kidney Foundation (Nierstichting) and the Dutch Kidney Patient Organization (NVN). The LONT centers have established the high quality NOTR (National Organ Transplant Registry) database for Kidney Transplantation. In recent years cooperation of the participating centers has markedly improved with successful projects such as the National Donor Exchange program marking this successful collaboration. The current development of national guidelines is another example of this improved collaboration. The sense of urgency to develop a national platform for high impact trials in kidney transplantation is mutual and the proposed study will be a starting point for a closer collaboration in future prospective clinical trials. To guarantee success of this trial also a large Belgian transplant center will participate.

2. OBJECTIVES

Primary Objective:
Part A: Primary endpoint: successful transplantation at two years after transplantation defined as: absence of graft or patient loss in the presence of an eGFR above 30 ml/min/1.73m^2.
Part B: Primary endpoint: successful transplantation at two years after transplantation defined as absence of graft or patient loss in the presence of an eGFR above 45 ml/min/ 1.73m^2.
Secondary Objective both parts:

- Incidence of individual endpoints of death, graft loss, rejection eGFR below 30 or 45 ml/min/ 1.73m^2 rejection at Months 12 and 24
- Rejection treatment and type of rejection treatment
- The evolution of renal function (eGFR) over time by slope analysis
- The incidence of adverse events, serious adverse events and adverse reactions
- The incidence of clinically relevant infections, new onset diabetes mellitus, malignancies and cardiovascular events
- Presence of frailty at 3, 12 and 24 months after transplantation and change in frailty from baseline
- Presence of markers for immunosenescence at 12 and 24 months and changes from baseline

- HRQoL at 0, 12 and 24 months and changes from baseline
- Development of donor specific antibodies

3. STUDY DESIGN

This is an open label, randomized, multicenter trial evaluating standard immunosuppression with Tacrolimus once daily (Envarsus®) and Mycophenolate Mofetil versus a low exposure Tacrolimus once daily (Envarsus®) regimen in combination with Everolimus.
The trial will consist of two parts:
Part A: Elderly recipients (≥65 years) of kidneys from elderly deceased donors (≥65 years) within the Eurotransplant Senior Program
Part B: Elderly recipients (≥65 years) of kidneys from living donors (all ages) or deceased donors (<65 years)
In part A, in each arm 96 patients will be included, in part B in each arm 90 patients, so in total **372** patients will be randomized within 24 hours after transplant surgery.
They will be randomized, both for part A as part B, to group 1 or group 2, in a 1:1 ratio.

Group 1: MMF + standard Tacrolimus once daily (Envarsus®)

Group 2: Everolimus + reduced Tacrolimus once daily (Envarsus®)

Group 1
Day 0 and day 4 Basiliximab 20 mg IV
Day 0 start prednisolone 20 mg bid, taper to 5 mg QD at M3, until M24
Day 0 start Tacrolimus once daily (Envarsus®) trough level 8-12, taper to trough level 5-8 at M6, until M24
or
When TAC is not tolerated: cyclosporine target trough level 150-200, taper to trough level 100-200 at M 6, until M24
Day 0 start MMF 500 mg bid until M24

Group 2
Day 0 and day 4 Basiliximab 20 mg IV
Day 0 start prednisolone 20 mg bid, taper to 5 mg QD at M3, until M24
Day 0 start Tacrolimus once daily (Envarsus®), trough level 5-7, taper to trough level 2-4 at M3, from M6 trough level 1.5-4 until M24
or
When Tacrolimus is not tolerated: cyclosporine target trough level 100-150, taper to 25-75 at M3, until M24
Day 0 start Everolimus, 3 mg bid, trough level 3-6 until M 24

See table 2.

The study will be scheduled in the following way:

Part A: Over the past three years (2014, 2015, 2016) 238 ESP transplantations have been performed in the Netherlands, thus a mean of 79 kidney transplantations per year in the ESP program. Very few patients in the ESP program will fall outside the inclusion criteria. With an expected consent rate of 70% (the consent rate of our recent trials) and the additional inclusion by a major Belgian transplantation center, it will be possible to achieve the inclusion target of 196 patients within 4 years. With an additional 2 years of follow-up the final data analysis will be possible within 6 years after initiating the study.

Part B: Over the past three years (2014, 2015, 2016) 291 living donor procedures and 193 deceased donor procedures with regular allocation (ETKAS) have been performed in recipients older than 65. Thus on a yearly basis a mean of 97 living donor procedures and 64 deceased donor procedures. In these groups 10 % of patients will not meet the inclusion criteria. With a consent rate of 70% and additional inclusion by a major Belgian transplantation center it will take 2 years to include the study population of 180 patients. With an additional 2 years of follow-up the final data analysis will be possible within 4 years after initiating the study.

Table 1 Visits and Assessments

	SCR/BL	Day 7	Week 4/6	Mo 3	Mo 6	Mo 9	Mo 12	Mo 18	Mo 24
Day	0	7	28/42	90	180	270	360	540	720
Time window				+/-7	+/-7	+/-14	+/-14	+/-21	+/-21
Randomization, IC	X								
Medical History	X								
Vital signs	X	X	X	X	X	X	X	X	X
Hematology	X		X	X	X	X	X	X	X
Biochemistry	X		X	X	X	X	X	X	X
Lipid Profile	X			X	X	X	X	X	X
Serology				X	X	X	X	X	X
Urine biochemistry: 24 H and spot	X		X	X	X		X	X	X
Trough levels (LCMS) CNI/EVR/MMF	X	X	X	X	X	X	X	X	X
Biobanking	X			X	X		X		X
DSA	X				X		X		X
Creatinine clearance				X	X	X	X		X
Gripstrength and Forms:	X						X		X
MOCA							X		X

Hematology: Hb, MCV, Ht, Leucocytes with differentiation, platelets

Serum biochemistry: Ureum, Creatinine, Sodium, Potassium, Albumin, Ca, P, fasting Glucose, HbA1c, Urate

Lipid profile: Fasting Cholesterol (Total, HDL and LDL) and Triglycerides

Urine biochemistry spot: Ureum, Creatinine, Sodium, protein, albumin

Urine 24 H: Protein, albumin

Serology serum **and urine**: EBV PCR, CMV PCR, BKV PCR

Biobanking: Plasma, serum, urine,
1 x EDTA 10 ml(4 x 1,5 ml) 1 x serum 10 ml(4 x 1,5 ml) 1 x spot urine (4 x 2 ml); 1 x PAXgene 2,5 ml (RNA), 3 x Lithium heparin 10 ml (PBMC isolation)

Forms: Fried
Clinical frailty scale
Short Physical Performance Battery
Comprehensive Geriatric Assessment
EQ-5D

MOCA: Montreal Cognitive Assessment

4. STUDY POPULATION

4.1 Population

Part A: Elderly recipients (≥65 years) receiving a kidney transplant from elderly deceased donors (≥65 years) within the Eurotransplant Senior Program (both DCD and DBD donors)

Part B: Part B: Elderly recipients (≥65 years) receiving a kidney transplant from either a living donor (all ages) or a deceased donor (<65 years) within regular Eurotransplant allocation (EKTAS)

4.2 Inclusion criteria

In order to be eligible to participate in this study, a subject must meet all of the following criteria:

Inclusion criteria

1. Written informed consent must be obtained before any assessment is performed
2. Male or female subject ≥ 65 years old
3. Subject randomized within 24 hours of completion of transplant surgery
4. Part A: Recipient of a primary (or secondary, if first graft is not lost due to immunological reasons) renal transplant from a deceased donor aged 65 years or older
5. Part B:Recipient of a primary (or secondary, if first graft is not lost due to immunological reasons) renal transplant from a deceased donor aged below 65 years or a living donor of any age

4.3 Exclusion criteria

A potential subject who meets any of the following criteria will be excluded from participation in this study

Exclusion criteria for both part A and B

1. Subject is a multi-organ transplant recipient
2. Recipient of ABO incompatible allograft or CDC cross-match positive transplant
3. Subject at high immunological risk for rejection as determined by local practice for assessment of anti-donor reactivity
4. Recipient of a kidney with a CIT > 24 hr
5. Recipients of a kidney from an HLA identical related living donor
6. Known intolerability for one or more of the study drugs
7. Subject who is HIV positive
8. HBsAg and/or a HCV positive subject with evidence of elevated LFTs (ALT/AST levels ≥ 2.5 times ULN). Viral serology results obtained within 6 months prior to randomization are acceptable

9. Recipient of a kidney from a donor who tests positive for human immunodeficiency virus (HIV), hepatitis B surface antigen (HBsAg) or anti-hepatitis C virus (HCV)
10. Subject with a BMI greater than 35
11. Subject with severe systemic infections, current or within the two weeks prior to randomization
12. Subject requiring systemic anticoagulation that cannot be temporarily interrupted and which would preclude renal biopsy
13. History of malignancy of any organ system (other than localized basal cell carcinoma of the skin), treated or untreated, within the past 5 years, regardless of whether there is evidence of local recurrence or metastases
14. Subject with severe restrictive or obstructive pulmonary disorders
15. Subject with severe hypercholesterolemia or hypertriglyceridemia that cannot be controlled
16. Subject with white blood cell (WBC) count ≤ 2,000 /mm3 or with platelet count ≤ 50,000 /mm^3

4.4 Sample size calculation

Part A: Based on an analysis of the NOTR with the current immunosuppressive regimen the proportion of elderly patients reaching the definition of successful transplantation is 45 % after two years after transplantation. With a combined low dose tacrolimus plus everolimus regimen a 14 % higher success rate of 59% is expected. To detect this difference with a power of 80% and an alpha of 0.05 in a 2-sided Z-test 96 patients will have to be included in each arm.
PART B : Based on an analysis of the NOTR with current immunosuppressive regimen the proportion of elderly patients reaching the defintion of succesful transplantation is 50% at two years after transplantation. With a combined low dose tacrolimus plus everolimus regimen a 14 % higher success rate of 64% is expected. To detect this difference with a power of 80% and an alpha of 0.05 in a 2-sided Z-test 90 patients will have to be included in each arm.

5. TREATMENT OF SUBJECTS

5.1 Protocol requested treatment

The following immunosuppressive drugs will be used in this study in accordance with this protocol.

Investigational and control immunosuppressive drugs:

- Basiliximab as 20 mg lyophilized vial for intravenous administration following reconstitution with sterile water, according to local practice
- Everolimus will be used as 0.25 and 0.75 mg tablets, or according to local practice
- MMF mycophenolate mofetil as 250 mg casules or 500 mg tablets, according to local practice
- Tacrolimus once daily (Envarsus®) as 0.75, 1.0 and 4.0 mg capsules, according to local practice.
- Corticosteroids for oral and i.v. administration will be used according to local practice

For induction therapy only basiliximab is allowed, rATG is allowed to be used as anti-rejection therapy.

Tacrolimus two times daily instead of tacrolimus once daily

Tacrolimus twice daily as 0.5, 1.0, 5.0 mg capsules can be used according to local practice if deemed indicated by the local investigator.

Cyclosporine administration

For all subjects who are unable to tolerate Tacrolimus, Cyclosporine can be administered, as capsules p.o., b.i.d., as 25, 50 and 100 mg capsules, according to local practice.
The lowest permitted dosing of Cyclosporine in this study is 25 mg b.i.d.
Cyclosporine trough levels should be adjusted to and maintained within the target ranges (see table 2):

Table 2

CNI trough target level ranges

Study visit	Tacrolimus ranges		Cyclosporine ranges	
	EVR arm	MMF arm	EVR arm	MMF arm
D1 until visit M3	5-7 ng/ml		100-150 ng/ml	
D1 until visit M6		8-12 ng/ml		150-200
From M3 - M24	2-4 ng/ml		25-75 ng/ml	
From M6 - M24	1.5-4 ng/ml	5-8 ng/ml		100-200

Treatment arms

Group 1: MMF + standard Tacrolimus once daily (Envarsus®)

Group 2: Everolimus + reduced Tacrolimus once daily (Envarsus®)

Patients will be treated as follows:

Group 1

Day 0 and day 4 Basiliximab 20 mg IV

Day 0 start prednisolone 20 mg bid, taper to 5 mg QD at M3, until M24

Day 0 start Tacrolimus once daily (Envarsus®), target trough level 8-12, taper to trough level 5-8 at M6, until M24 *or*

When TAC is not tolerated: cyclosporine target trough level 150-200, taper to 100-200 at M 6, until M24

Day 0 start MMF 500 mg bid until M24

Group 2

Day 0 and day 4 Basiliximab 20 mg IV

Day 0 start prednisolone 20 mg bid, taper to 5 mg QD at M3, until M24

Day 0 start Tacrolimus once daily (Envarsus®), target trough level 5-7, taper to trough level 2-4 at M3, trough level 1.5-4 from M6 until M24 *or*

When TAC is not tolerated: cyclosporine start level 100-150, taper to trough level 25-75 at M3, until M24

Day 0 start Everolimus, 3 mg bid, trough level 3-6 until M 24

5.2. Permitted dose adjustments and interruptions of study treatment

For all everolimus treated subjects who are unable to tolerate the protocol-specified dosing schedule, dose adjustments are permitted in order to keep the subject on investigational drug. The following guidelines should be followed, for management of lipid elevations refer to Section 5.3 for initial approach, prior to considering dose reduction. The everolimus dose should be decreased by at least 0.25 mg b.i.d if a dose reduction is necessary. If a temporary reduction in everolimus level is needed, the everolimus trough blood level should still be maintained no lower than 3 ng/mL. Everolimus should be discontinued if a trough level ≥3 ng/mL cannot be maintained due to toxicity. Severe and unremitting changes may also lead to investigational drug discontinuation.

For subjects having rATG administered, whether as induction therapy, DGF or anti-rejection treatment the relative impact of all medications to reduced hematological parameters should be considered, thus everolimus dose reduction to these guidelines may not always be necessary under these circumstances.

For subjects who are unable to tolerate Tacrolimus, Cyclosporine as 25, 50 and 100 mg capsules, can be used according to local practice.

If everolimus is interrupted for safety reasons for longer than 21 consecutive days study regimen should be discontinued. Everolimus may be interrupted during antibody treatment of rejection episodes. In case of any planned or emergency surgery during the study treatment period,

everolimus can be interrupted and a compensatory increase in CNI may be considered. In elective cases, this may be done 5 days before surgery which would allow restart of everolimus by 21 days post-surgery, in these cases everolimus must be re-introduced at the latest by day 27 after discontinuation.

For MMF, dose adjustment or interruption should follow local practice, and dose adjustments based on monitoring MMF concentrations are allowed. If MMF or either CNI is interrupted for more than 21 consecutive days the randomized study regimen must be discontinued. Subjects who discontinue their randomized study regimen are expected to remain in study with immunosuppression according to local practice until completing the study. All immunosuppressive therapy administered post discontinuation of study regimen (i.e. MMF+CNI or EVR+CNI) should be recorded on the concomitant Medications CRF under the immunosuppressive category.

For subjects having rATG administered, whether as induction therapy, DGF or anti-rejection treatment the relative impact of all medications to reduced hematological parameters should be considered, thus MMF dose reduction may not always be necessary under these circumstances. All dose changes must be recorded on the everolimus or MMF Dosage Administration Record CRF as appropriate.

Treatment of Acute Rejection episodes

In all suspected acute rejection episodes, regardless of initiation of anti-rejection treatment, an allograft biopsy must be performed within 48 hr. All episodes of acute rejection must be entered on the corresponding CRF (e.g. Acute Rejection CRF, Kidney Allograft Biopsy CRF, etc.) preferably within 24 hr.

Acute rejections should be treated with bolus methylprednisolone (other corticosteroids are acceptable at an equivalent dose) according to local practice. Recommended treatment is with at least 3 boluses of i.v. methylprednisolone with a minimal dose of 250 mg/bolus or at least 2 boluses of i.v. methylprednisolone with a minimal total dose of 750 mg.

Other anti-rejection therapies (i.e. antibody therapy) should only be used in cases of steroid-resistant rejections, vascular rejections or rejections with a Banff grade ≥ 2B.

All medications used for the treatment of suspected or confirmed acute rejections must be recorded on the Concomitant Medications CRF under the Immunosuppressive category.

Treatment of Delayed Graft Function (DGF)

In case of DGF, treatment will be according to local practice, but the randomized study regimen cannot be interrupted for more than 21 consecutive days. DGF treatments must maintain sufficient immunological coverage for the graft and may include maintaining, interrupting or reducing the dose of CNI and the use of anti-thymocyte globulin. In case of use of depleting

antibody hematological parameters (e.g. lymphocytes, WBC and platelets) must be monitored carefully. Use of MMF and everolimus in subjects randomized to the other treatment arm (i.e. everolimus or MMFrespectively) is prohibited during any interruption whether for DGF or other reasons.

If a subject with DGF is not able to return to or maintain their randomized study regimen as per protocol after 21 consecutive days of interruption, the subject should be discontinued from the study. If a subject is placed on permanent dialysis (or retransplanted) the Graft Loss, and Adverse Event CRFs should be completed, as well as an SAE report of Graft Loss submitted. Dialysis treatments should be recorded on the Dialysis CRF. Retransplantation should be recorded on the surgical and medical procedures CRF.

5.3 Concomitant Treatment

The investigator should instruct the subject to notify the study site about any new medications he/she takes after the subject was enrolled into the study. All medications, procedures and significant non-drug therapies (including physical therapy and blood transfusions) administered after the subject was enrolled into the study will be recorded on the Concomitant Medications CRF or the Surgical and Medical Procedures CRF respectively.

CMV prophylaxis

Cytomegalovirus (CMV) pre-emptive therapy and/or prophylaxis is recommended for all donor CMV positive/recipient CMV negative cases and considered for all recipients who are CMV positive. It is recommended that CMV prophylaxis be administered for a minimum of three months after transplantation. Prophylactic treatment with i.v. ganciclovir or oral valganciclovir is recommended and will be administered according to local practice, taking into account dose adjustments based on renal function. CMV prophylaxis is also recommended following antibody treatment of acute rejection episodes (see Section 5.2 above). Such prophylaxis should be recorded on the Concomitant Medications CRF.

***Pneumocystis jirovecii* (*Pneumocystis carinii*) pneumonia prophylaxis**

All subjects will be started on trimethoprim-sulfamethoxazole, starting when oral medication can be tolerated and continuing until at least six months post-transplant. After six months, subjects will be treated per local practice. Aerosolized pentamidine or dapsone may be administered to subjects who are unable to tolerate trimethoprim-sulfamethoxazole.

These prophylactic treatments above must be applied consistently across the study population at each given center to avoid bias and confounding in the results. Such prophylaxis should be recorded on the Concomitant Medications CRF.

Treatment of hyperlipidemia

During the course of the study, the lipid profile will be monitored. Lipid lowering medications should be administered according to guidelines and local practice.
The combination of HMG-CoA reductase inhibitors concomitantly with fibrates should be avoided, due to the increased risk of myopathy and rhabdomyolysis in combination with cyclosporine. If combination therapy is needed, ezetimibe should be used in combination with statins. Lipid lowering therapy should be optimized before dosage reduction of study medication is considered.

Hepatitis B (HBV) prophylaxis
Prophylaxis for recurrent hepatitis B during the course of this study is allowed and will be administered at the discretion of the investigator.

5.4 Prohibited Treatment

Prohibited Medication:
Sirolimus, Belatacept, Azathioprine.
Induction therapy with rATG, Campath or other nonprotocol antibody agents

5.5 Discontinuation of study treatment and discontinuation from study

The Dosage administration records for everolimus, MMF, tacrolimus and cyclosporine will be used to record if a subject has permanently discontinued study treatment and why.
Possible reasons for study treatment discontinuation are:
- Adverse Event
- Lack of Efficacy
- Technical problems
- Subject/Guardian Decision
- Lost to follow-up
- Death
- Graft Loss

The investigator should discontinue a subject from their randomized treatment regimen if, on balance, he/she believes that continuation would be detrimental to the subject’s well-being.

Subjects who discontinue their randomized study regimen (any component whether everolimus, MMF or the CNI) should remain in the study, if possible, and receive standard of care immunosuppression, according to local practice, until completing the study at Month 24. Visits and assessments for such subjects are described in Table 1. All immunosuppressants after discontinuation of study regimen must be recorded on the Concomitant Medication CRF under the Immunosuppressive category.

Subjects are expected to remain on the original CNI (combined with everolimus or MMF) to which they were randomized until at least Month 24. However, if subjects discontinue either CNI due to AE/tolerability and maintain everolimus or MMF combined with the alternate CNI, administered to respective target levels per protocol, they will not be considered to have discontinued study regimen/medication.

Discontinuation from Study and Study/Period completion

Subjects who discontinue their study treatment regimen should NOT be considered withdrawn from the study. Such subjects should remain in the study, if possible, and receive standard of care immunosuppression, according to local practice, until completing the study at Month 24. See Table 1 for the required assessments of these subjects after discontinuation of study regimen.

The status of every randomized subject must be recorded on Month 12 and/or Month 24 Study Phase Completion CRFs as either completing or discontinuing from the respective study period, with reason for discontinuation.

Possible reasons for discontinuation from the study are:

- Technical problems
- Subject/Guardian Decision
- Lost to follow-up
- Death
- Graft Loss
- Study terminated by sponsor

Subjects may voluntarily withdraw from the study for any reason at any time. They may be considered withdrawn if they state an intention to withdraw, fail to return for visits, or become lost to follow-up for any other reason.

If premature withdrawal occurs for any reason, the investigator must make every effort to determine the primary reason for a subject’s premature withdrawal from the study and record this information on the Study Phase Completion CRF at Month 12 and/or Month 24 as applicable.

For subjects who are lost to follow-up (i.e. those subjects whose status is unclear because they fail to appear for study visits without stating an intention to withdraw), the investigator must show "due diligence" by documenting in the source documents steps taken to contact the subject, e.g. dates of telephone calls, registered letters, etc. Subjects who are prematurely withdrawn from the study will not be replaced.

5.6 Randomization

The randomisation procedure will be designed and implemented by TCC using a web based system (ALEA). Every user will receive an individual login code with which they can randomise their patients.

- The web application will return the allocated treatment. As a confirmation the web application will also send an e-mail with the randomisation information to selected users The system is online 24 hours a day, 7 days a week

In case the system is not online and the randomisation of a patient cannot be delayed the researcher can manually randomise the patient. A procedure for manual randomisation will be sent to each researcher allowing to randomise patients.

6. METHODS

ECONOMIC EVALUATION

A cost-effectiveness analysis will be part of this project. Total treatment costs will be calculated based on observations in the trial, largely determined by short-term costs of medication, duration of hospitalization and dialysis. During the trial no major savings and health gains should be expected, however, an improvement of renal function at two years after transplantation will lead to a reduction of medical costs in the long run. Notably, improved eGFR within the trial will be extrapolated to long-term benefits of reduced hemodialysis and reduced medical costs. Additionally, a better renal function is associated with less complications and improved quality of life. These aspects will be integrated in a lifetime economic decision model.

6.1 Study procedures

All procedures will follow standard procedures, except biobanking. For this purpose extra blood samples will be withdrawn, 162,.5 ml per visit.

For geriatric, gastro-intestinal and QoL matters the next forms will be used:Fried, Clinical frailty scale , Short Physical Performance Battery, Comprehensive Geriatric Assessment, MOCA.

6.2 Withdrawal of individual subjects

Subjects can leave the study at any time for any reason if they wish to do so without any consequences. The investigator can decide to withdraw a subject from the study for urgent medical reasons.

6.3 Premature termination of the study

Termination of the study will occur at the discretion of the DSMB one year (or six months) after the inclusion of 100 patients.

7. SAFETY REPORTING

7.1 Section 10 WMO event

In accordance to section 10, subsection 1, of the WMO, the investigator will inform the subjects and the reviewing accredited METC if anything occurs, on the basis of which it appears that the disadvantages of participation may be significantly greater than was foreseen in the research proposal. The study will be suspended pending further review by the accredited METC, except insofar as suspension would jeopardize the subjects' health. The investigator will take care that all subjects are kept informed.

7.2 Adverse and serious adverse events

The Service Desk Clinical Research Office (UMCG) will be responsible to report (severe) adverse events on behalf of the coordinating investigator and the coordinating investigator will act as necessary to (serious) adverse events. Adverse events are defined as any undesirable experience occurring to a subject during the study, whether or not considered related to the investigational product. All adverse events reported spontaneously by the subject or observed by the investigator or his staff will be recorded.

A serious adverse event (SAE) is any untoward medical occurrence or effect that at any dose:

- results in death;
- is life threatening (at the time of the event);
- requires hospitalisation or prolongation of existing inpatients' hospitalisation;
- results in persistent or significant disability or incapacity;
- is a congenital anomaly or birth defect;
- is a new event of the trial likely to affect the safety of the subjects, such as an unexpected outcome of an adverse reaction, lack of efficacy of an IMP used for the treatment of a life threatening disease, major safety finding from a newly completed animal study, etc.

All SAEs will be reported through the web portal *ToetsingOnline* to the accredited METc that approved the protocol, within 15 days after the Service Desk Clinical Research Office (UMCG) has first knowledge of the serious adverse reactions. SAEs that result in death or are life threatening should be reported expedited. The expedited reporting will occur not later than 7 days after the coordinating investigator has first knowledge of the adverse reaction. This is for a preliminary report with another 8 days for completion of the report.

7.3 Suspected unexpected serious adverse reactions (SUSAR)

Adverse reactions are all untoward and unintended responses to an investigational product related to any dose administered.

Unexpected adverse reactions are adverse reactions, of which the nature, or severity, is not consistent with the applicable product information (e.g. Investigator's Brochure for an

unapproved IMP or Summary of Product Characteristics (SPC) for an authorized medicinal product).

The Service Desk Clinical Research Office (UMCG) on behalf of the coordinating investigator will report expedited the following SUSARs through the web portal *ToetsingOnline* to the METc:

- SUSARs that have arisen in the clinical trial that was assessed by the METc
- SUSARs that have arisen in other clinical trials with the same medicinal product, and that could have consequences for the safety of the subjects involved in the clinical trial that was assessed by the METc.

The remaining SUSARs are recorded in an overview list (line-listing) that will be submitted once every half year to the accredited METC. This line-listing provides an overview of all SUSARs from the study medicine, accompanied by a brief report highlighting the main points of concern. The expedited reporting of SUSARs through the web portal ToetsingOnline is sufficient as notification to the competent authority. The expedited reporting will occur not later than 15 days after the Service Desk Clinical Research Office (UMCG) has first knowledge of the adverse reactions. For fatal or life threatening cases the term will be maximal 7 days for a preliminary report with another 8 days for completion of the report.

7.4 Annual safety report

In addition to the expedited reporting of SUSARs, the Service Desk Clinical Research Office (UMCG) will submit on behalf of the coordinating investigator, once a year throughout the clinical trial, a safety report to the accredited METC. This safety report consists of:

- a list of all suspected (unexpected or expected) serious adverse reactions, along with an aggregated summary table of all reported serious adverse reactions, ordered by organ system, per study
- a report concerning the safety of the subjects, consisting of a complete safety analysis and an evaluation of the balance between the efficacy and the harmfulness of the medicine under investigation

7.5 Follow-up of adverse events

All adverse events will be followed until they have abated, or until a stable situation has been reached. Depending on the event, follow up may require additional tests or medical procedures as indicated, and/or referral to the general physician or a medical specialist.

7.6 Data Safety Monitoring Board (DSMB)

For this trial an independent data safety monitoring board (DSMB) has been formed. This committee will be guided by a charter defining their role and responsibilities, and methods specific to the committee. Its primary function will be periodic review of safety data, efficacy data,

and the events of interest. The Board will determine its own meeting frequency but will meet at least yearly face to face. The DMSB will advise the Steering Committee (SC) to continue, to adapt or to terminate the study. The SC will oversee the design, conduct and analysis of the study. The advices of the DSMB will be notified upon receipt by the Coordinating Investigator or Central Study Coordinator to the METc that approved the protocol. With this notification a statement will be included indicating whether the advice will be followed.

For this DSMB three expert, independent, national scientists and clinicians, who are not directly affiliated with the University Medical Center Groningen have been approached.
The DSMB will be composed of:

i. prof. dr. A.J. Hoitsma, retired internist-nephrologist
ii. dr. E.F. de Maar, internist-nephrologist, Wilhelmia Ziekenhuis Assen
iii. dr. A. Zwinderman, epidemiologist, AMC

Chair of the DSMB is prof. dr. A.J. Hoitsma

The DSMB members are knowledgeable on the subject but not have a direct interest in the research activities. Membership of the DSMB has been proposed and accepted for the duration of the study.
The advice(s) of the DSMB will only be sent to the sponsor of the study. Should the sponsor decide not to fully implement the advice of the DSMB, the sponsor will send the advice to the reviewing METc, including a note to substantiate why (part of) the advice of the DSMB will not be followed.

8. STATISTICAL ANALYSIS

The data from all centers will be pooled and summarized with respect to demographic and baseline characteristics and efficacy and safety observations. Exploratory analyses will be performed using descriptive statistics. Data will be presented for the complete intent-to-treat population (all patients having taken at least one dose of study medication) as well as the per-protocol population (all patients who completed the study without major protocol deviations). The assessment of safety will be based mainly on the frequency of adverse events, which includes all serious adverse events. Adverse events will be summarized by presenting the number and percentage of patients having any adverse event. Any other information collected (e.g. severity or relatedness to study medication) will be listed as appropriate.

9. ETHICAL CONSIDERATIONS

9.1 Regulation statement

This clinical study was designed and shall be implemented and reported in accordance with the ICH Harmonized Tripartite Guidelines for Good Clinical Practice, with applicable local regulations (including European Directive 2001/20/EC, US Code of Federal Regulations Title 21, and Japanese Ministry of Health, Labor, and Welfare), and with the ethical principles laid down in the Declaration of Helsinki.

9.2 Compensation for injury

The sponsor has an insurance which is in accordance with the legal requirements in the Netherlands (Article 7 WMO). This insurance provides cover for damage to research subjects through injury or death caused by the study.
The insurance applies to the damage that becomes apparent during the study or within 4 years after the end of the study.

10 ADMINISTRATIVE ASPECTS, MONITORING AND PUBLICATION

10.1 Monitoring and Quality Assurance

Monitoring Plan

Monitoring will be executed in compliance with the NFU (The Netherlands Federation of University Medical Centres)-guideline “Quality Assurance of research involving human subjects 2.0”. Monitoring for this study will be performed by the Service Desk Clinical Research Office (UMCG). Monitoring will be performed by an independent and qualified monitor.
To ensure patient’s rights, wellbeing and safety, compliance as well as quality of data, the monitors will visit the sites on a regular basis. For this study the risk classification is considered moderate (based on the NFU guideline), which implies intensive monitoring of at least 2 visits per site per year. Frequency of the visits depends on the actual patient inclusion rate and the observed events and deviations.
The monitor will verify the following items: patient flow (inclusion and dropout rate); Informed Consent Forms (presence, dates, signatures); Trial Master File and Investigator Site File (presence of all essential documents); in- and exclusion criteria; primary endpoint; SAEs / SUSARs (missed events, reporting procedures); study treatment (patient instructions, administration and accountability). 25% source data verification will be performed for all patients on a pre-selected set of data (focused on endpoints and safety). Source documents are defined as the patient’s hospital medical records, clinician notes, laboratory print outs, digital and hard copies of imaging, memos, electronic data etc.
The monitor will verify the compliance to study procedures, Standard Operating Procedures and other instructions. The presence of certificates, Standard Operating Procedures and instructions

related to devices, facilities, laboratories, pharmacy and other departments involved will be checked.

Findings from the monitoring visits will be reported by the monitor to the sponsor-investigator through a monitoring visit report. It is the responsibility of the sponsor-investigator to follow up on findings, deviations, queries or other issues where required.

10.2 Amendments

- A 'substantial amendment' is defined as an amendment to the terms of the METc application, or to the protocol or any other supporting documentation, that is likely to affect to a significant degree: the scientific value of the trial
- the conduct or management of the trial *or*
- the quality or safety of any intervention used in the trial

All substantial amendments will be notified to the METC and to the competent authority.

Non-substantial amendments will not be notified to the accredited METC and the competent authority, but will be recorded and filed by the sponsor.

10.3 Annual progress report

The sponsor/investigator will submit a summary of the progress of the trial to the accredited METc once a year. Information will be provided on the date of inclusion of the first subject, numbers of subjects included and numbers of subjects that have completed the trial, serious adverse events/ serious adverse reactions, other problems, and amendments.

10.4 Temporary halt and (prematurely) end of study report

The sponsor will notify the accredited METc and the competent authority of the end of the study within a period of 90 days. The end of the study is defined as the last patient's last visit.

The sponsor will notify the METc immediately of a temporary halt of the study, including the reason of such an action. In case the study is ended prematurely, the sponsor will notify the accredited METc and the competent authority within 15 days, including the reasons for the premature termination.

Within one year after the end of the study, the investigator/sponsor will submit a final study report with the results of the study, including any publications/abstracts of the study, to the accredited METc and the Competent Authority.

References:

1. Karim A. Recipient age and risk for mortality after kidney transplantation in england. *Transplantation*. 2014.

2. Meier-Kriesche HU.
Exponentially increased risk *Kidney Int*. 2001.

3. Tullius SG.
The combination of donor and recipient age is critical in determining host immunoresponsiveness and renal transplant outcome *Ann surg 252*. 2010.

4. Betjes M.
„Premature aging of circulating T cells in patients with end-stage renal disease." *kidney int.* 80, nr. 2 (2011): 208-17. *K I*. 2011.

5. Weng NP.
CD28(-) T cells: Their role in the age-associated decline of immune function *Trends Immunol*. 2009.

6. Dedeoglu B.
Loss of CD28 on peripheral T cells decreases the risk for early acute *PLos One*. 2016.

7. Peters-Sengers H. Stretching the limits of renal transplantation in elderly recipients. *J Am Soc Nephrol*. 2017.

8. Jacobson P.
Lower calcineurin inhibitor doses in older compared to younger kidney transplant recipients yield similar troughs *Am J Transplant*. 2012.

9. Naesens M.
The evolution of non-immune histological injury and its clinical relevance in adult-sized kidney grafts in pediatric recipients *Am J Transplant*. 2007.

10. Feutren G.

Risk factors for cyclosporine-induced ephropathy in patients with autoimmune diseases. international kidney biopsy registry of cyclosporine in autoimmune diseases *N Engll J Med*. 1992.

11. Qazi Y.

Efficacy and safety of everolimus plus low-dose tacrolimus versus mycophenolate mofetil plus standard-DoseTacrolimus in de novo renal transplant recipients *Am J Transplant*. 2016.

12. Langer RM.

Everolimus plus early tacrolimus minimization: A phase III, randomized, open-label, multicentre trial in renal transplantation *Transpl Int*. 2012.

13. Pascual J.

TRANSFORM: A novel study design to evaluate the effect of everolimus on long-termoutcomes after kidney transplantation *Open Access J Clin Trials*. 2014.
